# Extent of therapeutic support in Positive Psychotherapy: A randomized comparative efficacy study for the treatment of anxiety disorders in an online group setting

**DOI:** 10.1371/journal.pone.0354083

**Published:** 2026-07-28

**Authors:** Catiana L. Engelhardt, Sabrina Keller, Thomas Berger, Anton-Rupert Laireiter

**Affiliations:** 1 Department of Psychology, Paris Lodron Universität Salzburg, Salzburg, Austria; 2 Institute of Psychology, University of Bern, Bern, Switzerland; University of Anbar, IRAQ

## Abstract

**Objective:**

Positive Psychotherapy (PPT) is increasingly being studied as a potential treatment option for psychological diseases. In recent years the internet has been incorporated into traditional face-to-face therapy which, if successful in its treatment outcome, could lead to a more economic and affordable therapy since a certain proportion of therapy could be done without real-time therapist contact. The aim of this study is to investigate whether significant differences in outcome are found when comparing the effectiveness of PPT with complete therapeutic support (cPPT) in treating anxiety disorders compared to PPT with minimal therapeutic supervision (mPPT) in an online group setting. Participants in the cPPT group took part in ten weekly 120-minute online sessions with a therapist, while the mPPT group only had three 60-minute online sessions. For the remaining seven sessions, participants received videos. This paper presents the outcomes of both groups.

**Methods:**

This randomized controlled trial involved 106 participants allocated into either a group with complete therapeutic support (cPPT; *n* = 53) or a group with minimal supervision (mPPT; *n* = 53) for ten weeks. Data collection occurred through online questionnaires, evaluating anxiety levels, subjective happiness, quality of life, and psychological distress. These assessments were conducted at three time points: baseline (T0), post-intervention (T1), and three-month follow-up (T2).

**Results:**

Significant improvements over time were observed in both the cPPT and mPPT groups across several outcomes. Anxiety symptoms decreased substantially from pre-treatment to post-treatment and remained stable at the three-month follow-up. Significant time effects were found for fear and anxiety-related outcomes, including FQ ($\eta^{2}=0.10,\eta_{w}^{2}=0.32$), BAI ($\eta^{2}=0.17,\eta_{w}^{2}=0.42$), PAS ($\eta^{2}=0.14,\eta_{w}^{2}=0.39$), GAD-7 ($\eta^{2}=0.16,\eta_{w}^{2}=0.36$), with gains maintained at the three-month follow-up. Positive psychological outcomes also improved significantly over time. Significant effects were observed for subjective well-being and flourishing, including PPTI ($\eta^{2}=0.04,\eta_{w}^{2}=0.15$), FS ($\eta^{2}=0.02,\eta_{w}^{2}=0.07$), and SWLS ($\eta^{2}=0.04,\eta_{w}^{2}=0.14$). Psychological distress likewise decreased significantly across treatment. Significant time effects were found for ISR ($\eta^{2}=0.11,\eta_{w}^{2}=0.29$), PHQ-9 ($\eta^{2}=0.06,\eta_{w}^{2}=0.17$), with improvements remaining stable at follow-up. In all cases, there were no significant group × time interactions, indicating that the mPPT and cPPT groups showed comparable changes over time.

**Conclusion:**

Our study did not find significant differences between Positive Psychotherapy with minimal therapeutic supervision and PPT with complete therapeutic supervision. These findings challenge the traditional notion regarding the significance of face-to-face contact and therapist-patient interaction and suggest an economic and efficient alternative to the regular individual face-to-face therapy. As the participants were predominately female, further research should focus on a more gender-equal sample to generalize these findings.

## Introduction

Anxiety disorders are a global issue, with an estimated 4% of the earth’s population being affected and its prevalence continuing to rise [1]. Thus, providing various treatment applications among therapy approaches is necessary for good care. So far, face-to-face therapy has been the standard way of offering therapy. However, with the rise of the internet in mental health care spaces, and especially after the COVID-19 pandemic [2], technology has been increasingly used in therapy, whether it is video conferencing or using certain apps as an additional tool for the therapy. Many practitioners expressed the intention to continue using digital interventions or tools in addition to face-to-face therapy after the pandemic [2]. This approach, called blended therapy or blended psychotherapy, combines the classic face-to-face therapy with internet and/or mobile interventions [3,4]. This way of practice is viewed positively by practitioners as well as patients in recent studies [5,6]. The central goals of blended therapy are heightened efficiency and cost-effectiveness of mental health care [7]. So far, the efficacy and feasibility of blended therapy compared to a control group has been shown in studies using a range of mental disorders such as depression and anxiety disorders compared to a control group [7,8] as well as compared to face-to-face therapy [9]. As opposed to regular face-to-face therapy, blended therapy offers advantages for patients in several areas, such as bridging distance [10], more flexibility regarding time and location, and encouraging patients in practicing self-management [11]. Blended therapy can be implemented in an individual as well as in a group setting. Studies examining the efficacy of blended cognitive behavioral therapy showed that participants suffering from social phobia, panic disorder [12], or major depressive disorder [13] had the same positive therapy outcome as the traditional face-to-face CBT, with long-lasting effects [13]. The study by [9] showed that psychotherapy combined with web-based interventions – which according to [14] are primarily self-guided interventions carried out with the help of a prescribed online program operated via a website – yielded better results compared to regular psychotherapy for people with depression. A significant decrease has been found in the levels of depressive symptoms, anxiety symptoms as well as somatic symptoms and an increase in quality of life with small to medium between group effect sizes. Regarding blended group therapy, current literature is lacking however, positive outcomes have been observed when blended (psycho)therapy was implemented in a group setting. For example, in a study by [10] participants compared web-based interventions with treatment aspects such as group interaction or specific CBT interventions in terms of relevancy and 25% of the participants associated the utilization of the web-based interventions with treatment success. As of now, no studies have been made regarding blended positive psychotherapy in an individual or group setting for anxiety disorders. However, recent studies showed that people suffering from depression considered blended positive psychotherapy as a valuable treatment [15]. Slightly different from the traditional blended therapy application (face-to-face therapy mixed with web-based interventions), this study used the blended therapy approach in an online setting. This means that face-to-face therapy in this study commenced online in real-time. In other studies which used this approach [16,17], blended therapy succeeded, effectively treating the symptoms of depression. Our study examines whether the amount of therapeutic support has significant impact on the treatment outcome. Therefore, participants are divided into a group with complete therapeutic support and a group with minimal therapeutic support alongside the web-based interventions.

## Methods

The study aims to examine whether there are significant differences in outcomes over time between positive psychotherapy (PPT) delivered with complete therapeutic support (cPPT) and PPT delivered with minimal therapeutic support (mPPT) among individuals with anxiety disorders. For this purpose, we compared the two groups (cPPT and mPPT) with different levels of therapeutic support. The comparison of PPT with complete and minimal therapeutic support aims to emphasize the importance of the treatment setting. We investigated differences in the regularity, frequency and duration of treatment sessions and therapeutic superivsion. The group setting allowed an assessment of the exchange between patients and its impact on treatment outcome. In addition, the conditions for a good and successful therapeutic alliance between patient and therapist and their importance for the success of the therapy were examined. Furthermore, PPT was examined not only for its short-term effects, but also for its medium-term effects. As the demand for psychotherapy/psychological interventions exceeds the supply, efficient and economical ways must be found to serve more patients. Therefore, the study focuses on the effectiveness and efficiency of PPT in an online setting.

### Study design and protocol

**Fig 1:**
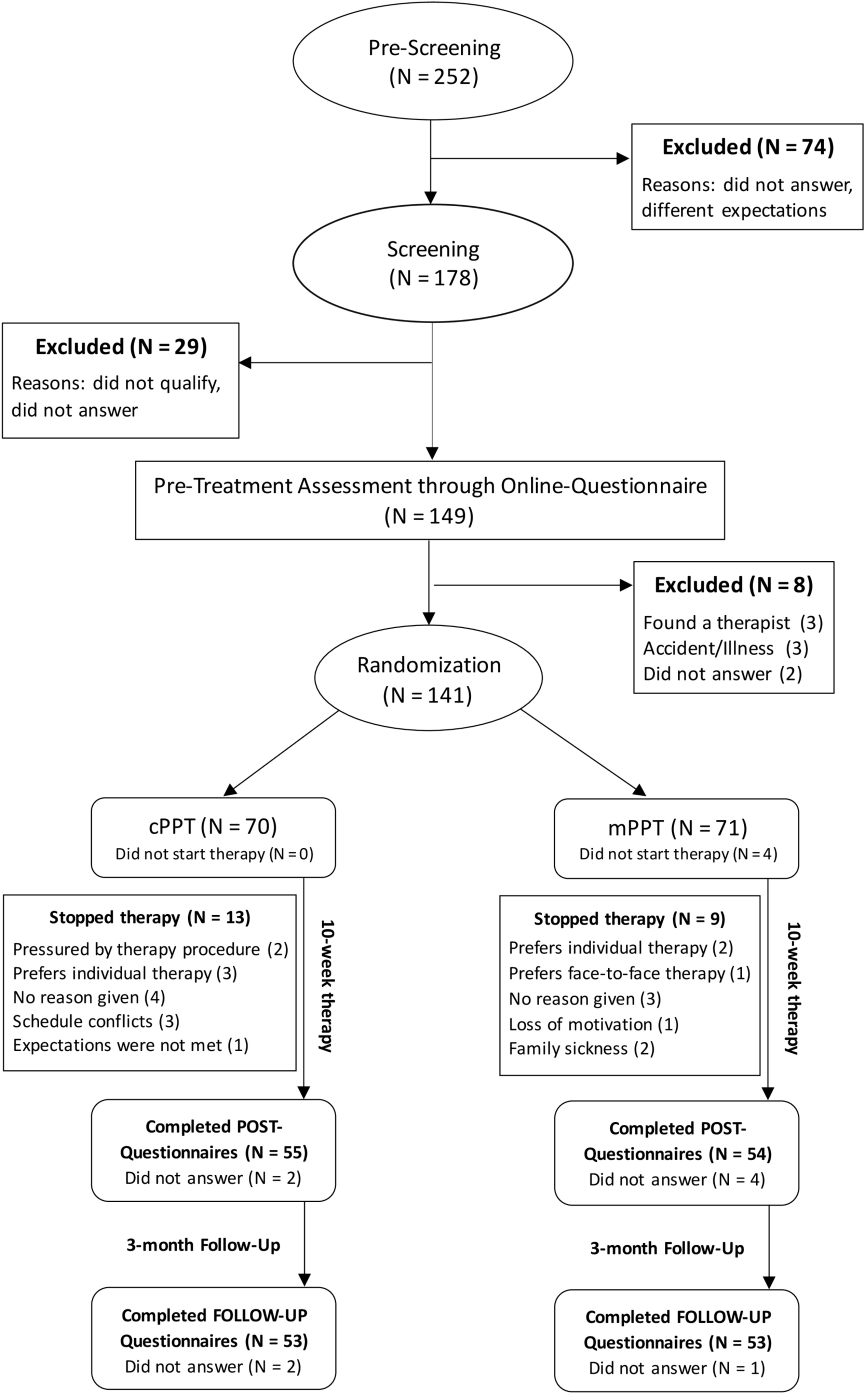
Flowchart of the study design.

The study presented here, is part of a larger series of studies initially described in the study protocol [18] in which a three-arm randomized controlled trial comparing CBT, cPPT (complete therapeutic supervision), and mPPT (minimal therapeutic supervision) was conducted. This manuscript focuses on the comparison between the cPPT and mPPT groups. The study procedure is shown in Fig 1. This study is a randomized controlled trial with two conditions: the PPT group with complete therapeutic support (cPPT) and the PPT with minimal therapeutic support (mPPT). The participants were randomly assigned to both groups using the online platform randomizer.org. Neither the participants nor the practitioners had the authority to choose group allocation. The treatment took place over a period of ten weeks in an online group setting. The participants completed the same questionnaires at three measurement points: before the start of the study (PRE measurement, T0), after completion of therapy (POST measurement, T1), and at the follow-up examination three months after the end of treatment (FOLLOW-UP measurement, T2). The study protocol was approved by the ethic committee and is provided as supporting information, with the original German version accessible under S1 Protocol and the English translation under S2 Protocol. The study is registered with the German Clinical Trial Register under the number DRKA00027521. Ethical approval for this study series was granted by the Ethics Committee of Paris Lodron University Salzburg (Approval No. EK-GZ 22/2021). Verbal informed consent was obtained from all participants for the use and publication of anonymized data. The Ethics Committee approved verbal consent as appropriate for the online study format, where participants were not physically present and participation posed minimal risk. All procedures were conducted in accordance with the Declaration of Helsinki. Participant data were fully anonymized before analysis to ensure privacy and confidentiality. No animals were involved in this study. Additionally, the CONSORT checklist is available as supporting information under S3 Appendix. The complete raw data collected during the study are available as supporting information file under S4 Data.

#### Inclusion and exclusion criteria.

Inclusion criteria comprised individuals aged 18–65, proficiency in the German language, and a diagnosis of F41.0 panic disorder with or without agoraphobia and/or F41.1 generalized anxiety disorder and/or F40.1 social phobia according to ICD-10. Exclusion criteria were concurrent or planned participation in psychological or psychotherapeutic interventions within the next three months, as well as the presence of a major depressive episode, bipolar affective disorder or mania (current or previous), schizophrenic or schizoaffective disorder, grief reaction, severe anorexia or bulimia, substance dependence (alcohol, illicit drugs), severe personality disorder, acute suicidality. While the use of psychotropic drugs was not an exclusion criterion, recent changes in medication, dosage, or complete discontinuation within the past or upcoming three months were considered.

#### Participant recruitment process.

Recruitment initiated with the distribution of flyers in various locations, including pharmacies, doctors’ and psychotherapists’ offices, as well as public spaces like universities, supermarkets, and churches. Social media platforms such as Facebook and Instagram were also utilized for study promotion. The recruiting process began on March 8^th^, 2022 and ended on November 3^rd^, 2022. Interested individuals underwent an initial online questionnaire pre-screening, followed by a screening appointment conducted via video call for those who passed the pre-screening. Inclusion and exclusion criteria, along with diagnostic assessments, were determined using the German versions of the Diagnostic Interview in Psychological Disorders (MINI-DIPS; [19]) and the Personality Disorder Screening – Short Form (PSS-K; [20]). Participants received detailed information about the study process, content, and data collection procedures and had to give their verbal consent as a prerequisite for participation in the study. Verbal consent was giving during screening and documented with the date and time.

#### Study sample.

As outlined in [18], the number of participants was determined with the power analysis tool G*Power 3.1. Since in this article the comparison between the cPPT and the mPPT is presented, the sample size is reduced to 106. The initial post-screening number of 141 was reduced due to dropouts (see Fig 1). The study primarily included female participants, with 103 out of the 106 total participants (97.2%) being women.

Table 1 presents the demographic profiles along with the primary and secondary outcome measures of the study sample at baseline.

**Table 1:** Descriptive statistics for demographic, outcome variables, and significance tests at baseline.

	cPPT	mPPT	Total	
	(n=53)	(n=53)	(n=106)	Statistics
**Demographics**				
Gender: *n*(%)	Female: 52 (98.1)	Female: 51 (96.2)	Female: 103 (97.2)	$\chi^{2}$(1)=0.343, p = 1.000
Age: Mean (SD)	34.62 (8.22)	34.32 (10.83)	34.47 (9.83)	t(104) = 0.157, p = 0.875
				
**Primary outcomes**				
**Level of anxiety**				
FQ	21.64 (9.30)	18.47 (9.27)	20.06 (9.38)	t(104) = 1.757, p = 0.082
GAD7	12.98 (4.08)	11.92 (3.87)	12.45 (4.00)	t(104) = 1.367, p = 0.175
BAI	27.96 (11.20)	25.96 (8.98)	26.96 (10.15)	t(104) = 1.014, p = 0.313
PAS	22.92 (9.90)	21.23 (9.06)	22.07 (9.49)	t(104) = 0.921, p = 0.359
**Subjective experience of happiness**				
PPTI	86.92 (14.26)	89.30 (15.56)	88.11 (14.90)	t(104) = -0.820, p = 0.414
FS	38.64 (8.87)	38.81 (8.02)	38.73 (8.42)	t(104) = -0.103, p = 0.918
SWLS	20.28 (6.37)	19.66 (7.58)	19.97 (6.98)	t(104) = 0.458, p = 0.648
				
**Secondary outcomes**				
**General psychological distress**				
ISR	46.08 (14.88)	43.81 (14.12)	44.94 (14.48)	t(104) = 0.804, p = 0.423
PHQ9	11.49 (5.41)	10.79 (5.36)	11.14 (5.37)	t(104) = 0.667, p = 0.506

### Interventions

The treatment manuals and therapeutic materials for the cPPT group and the mPPT shared identical content. The distinction between both groups lays in the level of support provided by therapists and the setting. In the cPPT group, participants engaged in weekly 120-minute online sessions with a therapist, adhering to the manual. The mPPT group used the same workbook and covered the same topics, but had three (first, fifth, and tenth sessions) 60-minute online sessions with a therapist. For the remaining seven sessions, participants received concise video recordings explaining the content and homework, with optional therapeutic support via email. Both groups were organized into coworking couples at the beginning of therapy, maintaining the pairing unless there was a reason for change (e.g., dropout). Within these pairs, participants discussed weekly homework assignments using their preferred communication medium (e.g., video call, phone call, face-to-face). The PPT manual, based on the official manual by [21], was adapted for the study to include ten sessions and incorporate exercises from the study leaders’ PPT repertoire. During the ten weeks, the participants were encouraged to recognize and increase experiences of happiness and gratitude. They learned about the value of character strengths and signature strengths and ways to use them as an advantage in daily challenging situations. Self-compassion was practiced by being mindful and meditating. Furthermore, individual resource kits and goals were made to leave the patients material and guidelines for after the end of treatment. Both groups had access to a website with instructional videos and worksheets, which were identical for both groups. Due to ethical considerations, we did not compare to a waiting list control group, as this would withhold needed therapeutic support from the patients.

### Outcomes measures

The outcome measures are divided into primary outcome variables and a secondary outcome variable. The primary outcome measures are the extent of anxiety (BAI, PAS, GAD-7, FQ) and subjective experiences of happiness (PPTI, FS, SWSL), while the secondary outcome measure is the level of general psychological distress (ISR & PHQ-9). All outcome variables are based on self-reports and were tested using the following questionnaires.

The Beck Anxiety Inventory, German version (BAI; [22]) assesses the level of anxiety in adolescents and adults experienced over the past seven days (Cronbach’s α=.91; [23]; high correlation (*r* = .64)) with the Hamilton Anxiety Rating Scale (HAM-A; [24]). The questionnaire has 21 items and a four-point Likert scale. The Panic and Agoraphobia Scale, German version (PAS; [25]) measures the severity of the patient’s symptoms in the past seven days regarding five aspects that affect quality of life (e.g., panic attacks, agoraphobic avoidance, health concerns) with 13 items (Cronbach’s α=.86; high correlation of *r* = .91 with the Clinical Global Impression Scale (CGI; [25]). The Generalized Anxiety Disorder 7, German version (GAD-7; [26]) evaluates the frequency of the anxiety symptoms regarding the last two weeks concisely by using only seven items (Cronbach’s α=.92; high correlation with the BAI *r* = .72). The Fear Questionnaire, German version (FQ; [27]) contains five items regarding the severity of avoidance behavior in situations that are anxiety inducing for the test-taker (Cronbach’s α is moderate; all scales showed moderate construct validity [28]).

The Positive Psychotherapy Inventory, German version (PPTI; [21]) is a comprehensive questionnaire measuring the five aspects of well-being based on the PERMA theory of well-being (e.g., positive emotions, engagement, relations, meaning, achievement; (Cronbach’s α=.89; convergent validity of *r* between .50 and .70 with the Ryff’s Psychological Well-Being Scale (PWB)). The Flourishing Scale, German version (FS; [29]) is an instrument assessing subjective psychological well-being (Cronbach’s α=.79; strong correlation of *r* = .57 with the Short-Form Health Survey (SF-12; [30]).

The Satisfaction with Life Scale, German version (SWLS; [31]) measures one’s satisfaction with life using five items (Cronbach’s α=.87; [32]; moderate correlation of *r* = .46 with the Life Satisfaction Index; [31]). A self-designed questionnaire was used for an external (third-party) assessment by the therapists on the extent of anxiety (regarding tension, anxious mood, anxiety, sleep disturbance, depressiveness, anxious behavior, physical symptoms, psychological stress, impairment of lifestyle, life satisfaction, motivation & energy). Questions such as “To what extent does the patient show/report an anxious mood?” and “How impaired does the patient appear to you in his lifestyle due to his psychological stress?” were used to assess the above-mentioned topics. Questionnaires such as the PPTI, the FS, and the SWLS were used as inspiration when creating the test items.

The ICD-10 Symptom Rating, German version (ISR; [33]) evaluates the severity of the psychological symptoms such as depression, anxiety, and somatization with 29 items (test-retest reliability on clinical samples of *r* = .94 [34] and proven validity [33]). The Patient Health Questionnaire, German version (PHQ 9; [35]) consists of nine questions regarding depression using a four point Likert scale. Each item represents one of the nine DSM-IV criteria for the diagnosis “Major depression” (Cronbach’s α=.88; [36] and test sensitivity of = .80 when validated against major depressive disorder; [37]).

### Data collection and security protection

Participants completed all self-report questionnaires for T0, T1, and T2 online using Google Forms. Data access was restricted to study personnel and investigators. Anonymity was safeguarded at the practitioner level by adherence to professional confidentiality laws (Austrian Psychologists’ Act and German Psychotherapists’ Act). At the research level, individual codes were assigned to each subject, ensuring data remained non-identifiable. Personal data is stored in a password-protected table on a secure laptop.

### Statistical analysis

Initially, a descriptive analysis was conducted, presenting the means and standard measurement of the treatment condition (cPPT) and the control group (mPPT) for each dependent variable across the three measurement points (pre, post, follow-up). Additionally, baseline comparisons were made for demographic variables such as age and gender, as well as for each dependent variable measuring anxiety levels (FQ, GAD, BAI, PAS), subjective happiness (FS, SWLS, PPTI), and general psychological distress (ISR, PHQ9). A *t*-test or $\chi^{2}$-test was used for each variable to identify significant baseline differences. Furthermore, the data was examined separately for each pair of therapists within the treatment group to explore differences between the clusters. Each individual in the mPPT group received treatment from the same therapist, thus the control group was treated as a single cluster [38]. As is to be expected for these studies, we experienced drop-outs in both groups. We restricted our analyses to completers only. In an additional investigation, we found that non-completers did not differ significantly with respect to group assignment and baseline levels of the dependent variables. The investigation of whether the cPPT and mPPT groups changed differently over time was conducted using linear mixed-effects models. Separate linear mixed-effects models were estimated for each outcome variable. Random intercepts for subjects were included to account for the repeated-measures structure of the data. To obtain more accurate statistical inference in mixed-effects models, Satterthwaite-adjusted degrees of freedom were applied for all fixed effects tests. The analyses were conducted using the R packages lme4 and lmerTest [39]. Effect sizes were estimated using the approach proposed by [40] for calculating $\eta^{2}$ and $\eta_{w}^{2}$. To control for multiple testing, *p*-values were adjusted separately within predefined families of theoretically related outcomes using the Benjamini-Hochberg false discovery rate (FDR) correction procedure. The use of hypothesis families followed recommendations by [41] regarding the control of false rejections across related sets of hypotheses.

## Results

### Descriptive statistics

Table 2 provides a detailed summary of the pre, post, and follow-up means along with the corresponding standard deviations for primary outcomes related to anxiety (FQ, GAD, BAI, PAS) and subjective experience of happiness (FS, SWLS, PPTI), as well as the secondary outcome of general psychological distress in both the cPPT group and mPPT group. Upon examining the means, a significant difference between pre- and post-measurements is evident in the treatment PPT group for all nine outcome variables. A similar difference is observed in the mPPT. However, there is no notable difference between the post-intervention and follow-up periods within each group.

**Table 2:** Means and standard deviations for all outcome variables in pre-, post-, and follow-up measurement points, and range of questionnaires.

		Pre		Post		Follow-up		
	Group	M	SD	M	SD	M	SD	Range
**Primary outcomes**								
**Anxiety**								
FQ	cPPT	21.64	9.30	15.34	9.91	14.77	9.91	0-120
	mPPT	18.47	9.27	11.70	8.79	11.25	9.33	
GAD	cPPT	12.98	4.08	9.36	4.44	9.00	5.10	0-21
	mPPT	11.92	3.87	7.85	4.66	7.19	4.25	
BAI	cPPT	27.96	11.20	19.17	12.65	17.17	11.54	0-63
	mPPT	25.96	8.98	15.51	10.20	14.53	9.60	
PAS	cPPT	22.92	9.91	15.74	9.43	15.40	10.42	0-52
	mPPT	21.23	9.06	12.58	9.67	11.92	9.35	
**Subjective experience of happiness**								
FS	cPPT	38.64	8.87	41.02	8.60	40.77	9.00	8-56
	mPPT	38.81	8.03	42.00	10.92	43.13	10.40	
SWLS	cPPT	20.28	6.37	22.17	7.19	22.70	6.01	5-35
	mPPT	19.66	7.58	23.36	7.18	23.45	7.74	
PPTI	cPPT	86.92	14.26	93.26	13.53	91.32	17.50	25-125
	mPPT	89.30	15.56	97.53	16.86	96.21	19.14	
**Secondary outcomes**								
**General psychological distress**								
ISR	cPPT	46.08	14.88	35.75	15.99	34.79	18.61	0-116
	mPPT	43.81	14.12	31.36	16.67	29.81	17.28	
PHQ9	cPPT	11.49	5.41	9.06	5.07	9.66	5.98	0-27
	mPPT	10.79	5.36	7.55	4.94	7.34	4.66	

### Clustered by therapists

**Fig 2:**
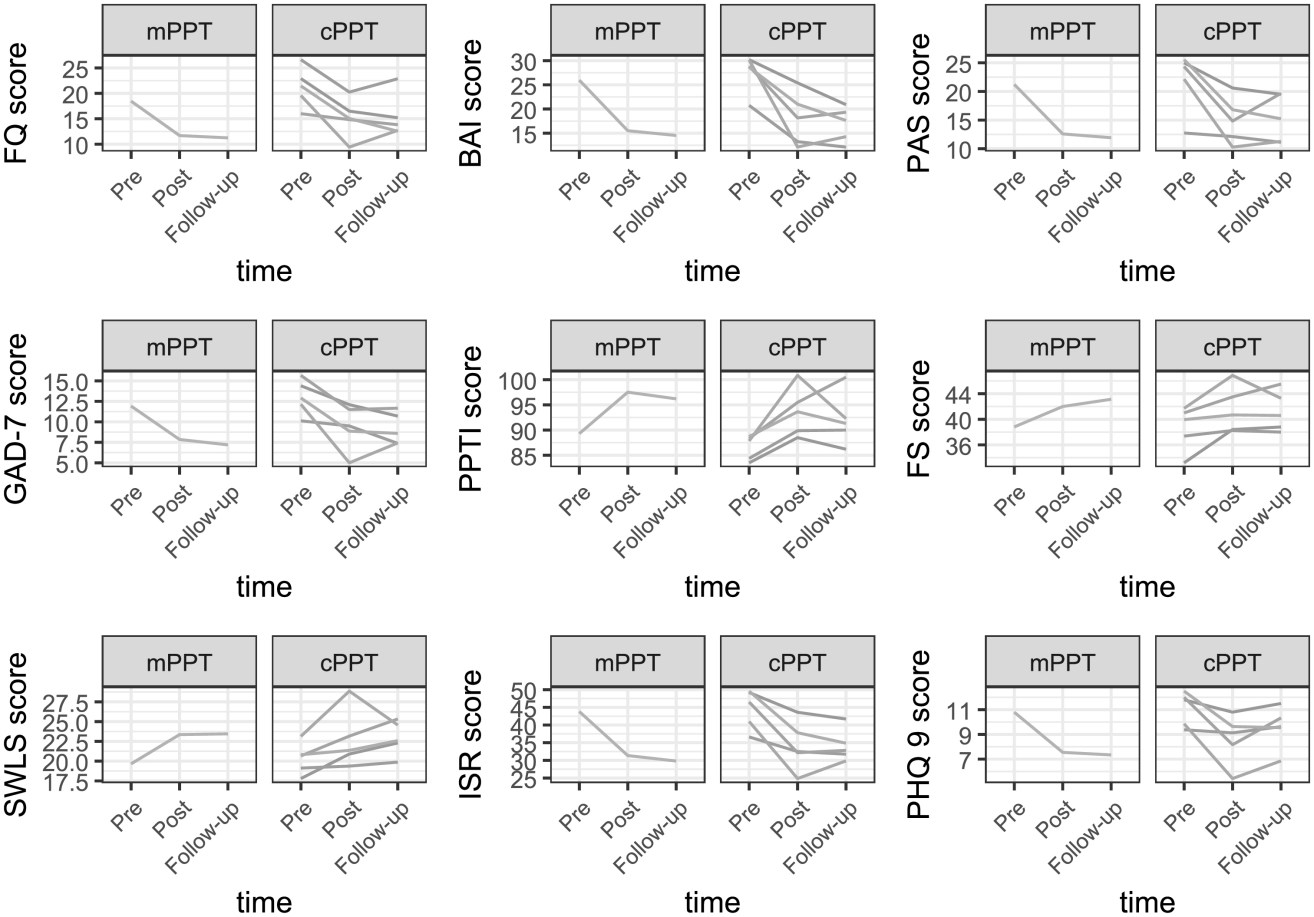
Behavior of each therapist cluster over time for each outcome variable.

Participants in the cPPT condition were treated by different pairs of therapists. In total, there were five therapeutic pairs handling the cPPT condition, with each cluster comprising between six and 22 individuals. The mPPT was treated as a single cluster of 53 individuals. Fig 2 presents the graphical analysis, showing the behavior of each cluster over time for each outcome variable.

The graphic indicates distinct starting points for each cluster in the cPPT condition across most outcome variables, but little variation in their trajectories over time. While the intercepts differ noticeably, the slopes exhibit consistent patterns with minimal deviation. This consistency suggests a high degree of similarity in temporal trends among the clusters.

### Primary outcomes

#### Level of anxiety.

The linear mixed-effects model analyses revealed no statistically significant group × time interactions for any of the primary anxiety outcomes. The interaction effects were non-significant for the Fear Questionnaire (FQ) ($F(2,208)=0.05,p=\mathrm{.952},\eta^{2}=0.00,\eta_{w}^{2}=0.00$); the Beck Anxiety Inventory (BAI) ($F(2,208)=0.38,p=\mathrm{.915},\eta^{2}=0.00,\eta_{w}^{2}=0.00$), the Panic and Agoraphobia Scale (PAS) ($F(2,208)=0.68,p=\mathrm{.915},\eta^{2}=0.00,\eta_{w}^{2}=0.00$), and Generalized Anxiety Disorder 7 (GAD7) ($F(2,208)=0.39,p=\mathrm{.915},\eta^{2}=0.00,\eta_{w}^{2}=0.00$). These findings are detailed in Table 3. Accordingly, participants in the cPPT group did not differ significantly from those in the mPPT group regarding changes in fear and anxiety symptoms over time. However, the main effect of time was significant for all primary outcomes, indicating overall improvement regardless of treatment condition.

**Table 3:** *F*-tests for FQ, BAI, PAS, and GAD-7.

	FQ						BAI						PAS						GAD7					
Factor	df1	df2	*F*	*p*	$\eta^{2}$	$\eta_{w}^{2}$	df1	df2	*F*	*p*	$\eta^{2}$	$\eta_{w}^{2}$	df1	df2	*F*	*p*	$\eta^{2}$	$\eta_{w}^{2}$	df1	df2	*F*	*p*	$\eta^{2}$	$\eta_{w}^{2}$
group	1	102.00	4.36	0.085	0.03	–	1	102.00	2.31	0.131	0.01	–	1	102.00	2.72	0.131	0.02	–	1	102.00	4.23	0.085	0.03	–
time	2	208.00	50.39	<0.001	0.10	0.32	2	208.00	78.32	<0.001	0.17	0.42	2	208.00	67.86	<0.001	0.14	0.39	2	208.00	61.00	<0.001	0.16	0.36
group:time	2	208.00	0.05	0.952	0.00	0.00	2	208.00	0.38	0.915	0.00	0.00	2	208.00	0.68	0.915	0.00	0.00	2	208.00	0.39	0.915	0.00	0.00
gender	1	102.00	2.79	0.333	0.02	–	1	102.00	0.11	0.946	0.00	–	1	102.00	1.94	0.333	0.01	–	1	102.00	0.00	0.946	0.00	–
age	1	102.00	0.40	0.577	0.00	–	1	102.00	1.92	0.337	0.01	–	1	102.00	8.32	0.019	0.06	–	1	102.00	0.31	0.577	0.00	–

The FQ scale showed no significant main effect of group, $F(1,102)=4.36,p=\mathrm{.085},\eta^{2}=0.03$, indicating that overall fear levels did not differ significantly between the cPPT and mPPT groups. However, there was a significant main effect of time on fear ($F(2,208)=50.39,p<\mathrm{.001},\eta^{2}=0.10,\eta_{w}^{2}=0.32$). The time effect accounts for 10% of the total variation and for 32% of the variance within participants. Pairwise comparisons revealed that participants had significantly lower fear levels at both post-treatment (diff=6.54,t(208)=8.35,p<.001) and follow-up (diff=7.05,t(208)=9.00,p<.001) compared to pre-treatment. The participants in the study had 6.54- and 7.05-points lower levels of fear in the post and follow-up measurement compared to pre. However, the interaction between group and time was not statistically significant ($F(2,208)=0.05,p=\mathrm{.952},\eta^{2}=0.00,\eta_{w}^{2}=0.00$), indicating that the reduction in fear levels over time did not differ between the cPPT and mPPT groups.

Regarding the BAI, there was no significant group effect ($F(1,102)=2.31,p=\mathrm{.131},\eta^{2}=0.01$), and no significant interaction between group and time ($F(2,208)=0.38,p=\mathrm{.915},\eta^{2}=0.00,\eta_{w}^{2}=0.00$), indicating that the reduction in anxiety symptoms over time did not differ between the cPPT and mPPT groups. However, the main effect of time was significant ($F(2,208)=78.32,p<\mathrm{.001},\eta^{2}=0.17,\eta_{w}^{2}=0.42$). The main effect of time accounts for 17% of the total variation and for 42% of the variance within participants in BAI. Pairwise comparisons revealed that participants had significantly lower anxiety scores at both post-treatment (diff=9.62,t(208)=9.98,p<.001) and follow-up (diff=11.11,t(208)=11.53,p<.001) compared to pre-treatment.

In the PAS, neither the main effect of group ($F(1,102)=2.72,p=\mathrm{.131},\eta^{2}=0.02$) nor the interaction between group and time was significant ($F(2,208)=0.68,p=\mathrm{.915},\eta^{2}=0.00,\eta_{w}^{2}=0.00$). These findings indicate that changes in panic and agoraphobia symptoms over time did not differ significantly between the cPPT and mPPT groups. The main effect of time, however, was statistically significant ($F(2,208)=67.86,p<\mathrm{.001},\eta^{2}=0.14,\eta_{w}^{2}=0.39$). The effect accounts for 14% of the total variation and for 39% of the variance within participants in PAS. Pairwise comparisons showed significantly lower PAS scores at post-treatment (diff=7.92,t(208)=9.77,p<.001) and follow-up (diff=8.42,t(208)=10.38,p<.001) compared to pre-treatment.

Similarly, for the GAD-7, there was no significant main effect of group ($F(1,102)=4.23,p=\mathrm{.085},\eta^{2}=0.03$), and no significant group × time interaction ($F(2,208)=0.39,p=\mathrm{.915},\eta^{2}=0.00,\eta_{w}^{2}=0.00$), indicating that both groups improved similarly over time. A significant main effect of time was found ($F(2,208)=61.00,p<\mathrm{.001},\eta^{2}=0.15,\eta_{w}^{2}=0.36$), with pairwise comparisons showing significantly lower GAD-7 scores at post-treatment (diff=3.85,t(208)=8.92,p<.001) and follow-up (diff=4.36,t(208)=10.10,p<.001) compared to pre-treatment. The GAD-7 results show that time accounted for 15% of the total variation and 36% of the within participants variance.

**Fig 3:**
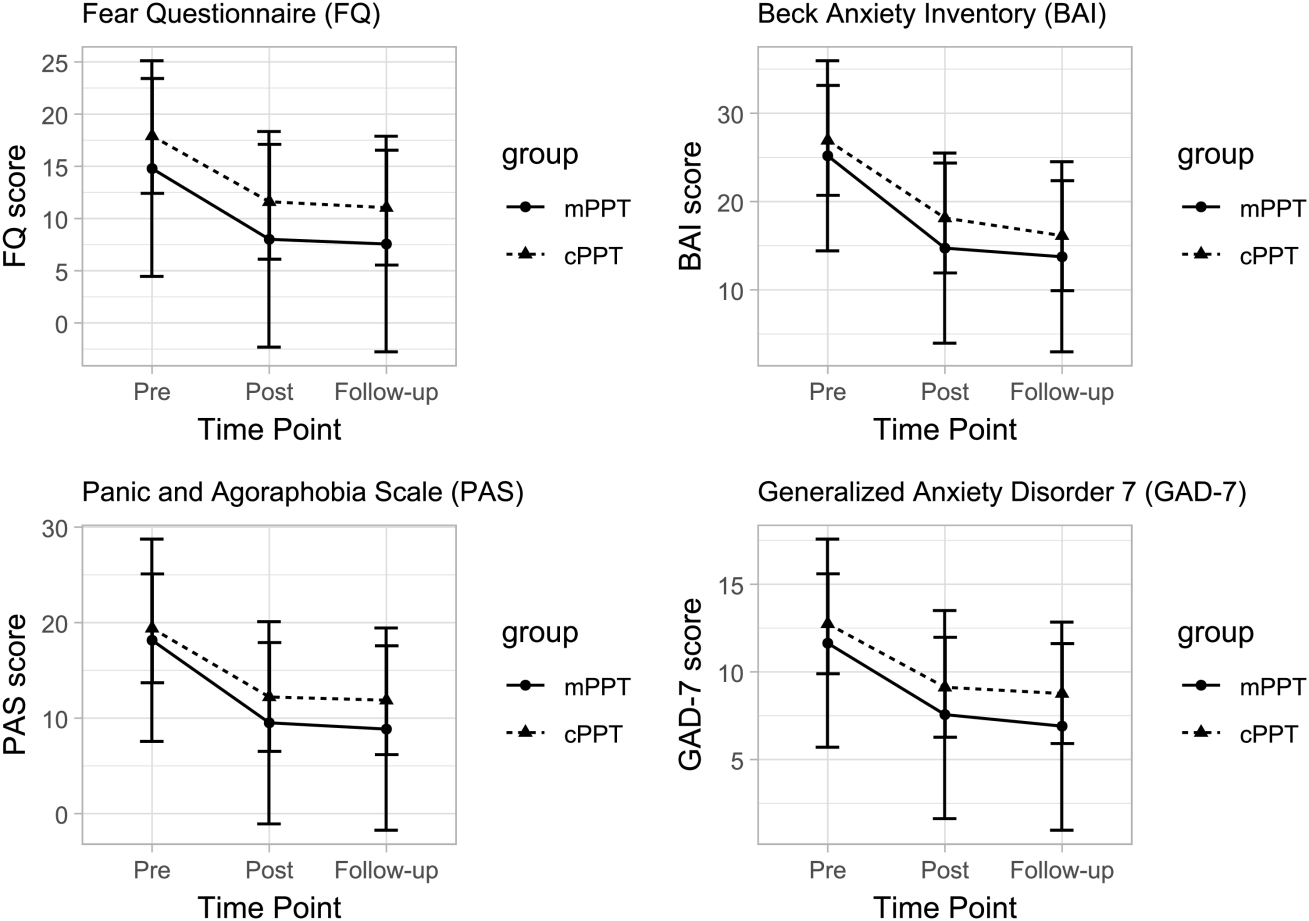
Plots of means for FQ, BAI, PAS, and GAD-7.

No significant interaction effects were noted in any of these outcomes. However, within each outcome variable, there was a notable and significant decrease in the outcome measure between pre-post and pre-follow-up measurements for both treatment groups. All time effects showed medium to large effect sizes, with $\eta^{2}$ ranging from .10 to .17 and $\eta_{w}^{2}$ ranging from .32 to .42. All results are adjusted for gender and age. All results are adjusted for gender and age. A comprehensive summary of the LMM results is presented in Tables 3 and 4 and Fig 3.

**Table 4:** Pairwise comparison for time for FQ, BAI, PAS, and GAD-7.

	FQ					BAI					PAS					GAD7				
Contrast	Est	SE	df	*t*	*p*	Est	SE	df	*t*	*p*	Est	SE	df	*t*	*p*	Est	SE	df	*t*	*p*
pre-post	6.54	0.78	208.00	8.35	<0.001	9.62	0.96	208.00	9.98	<0.001	7.92	0.81	208.00	9.77	<0.001	3.85	0.43	208.00	8.92	<0.001
pre-(follow-up)	7.05	0.78	208.00	9.00	<0.001	11.11	0.96	208.00	11.53	<0.001	8.42	0.81	208.00	10.38	<0.001	4.36	0.43	208.00	10.10	<0.001
post-(follow-up)	0.51	0.78	208.00	0.65	0.792	1.49	0.96	208.00	1.55	0.272	0.50	0.81	208.00	0.62	0.811	0.51	0.43	208.00	1.18	0.466

#### Subjective experience of happiness.

The analysis of indicators related to the subjective experience of happiness, conducted through a linear mixed model, aligns with the findings regarding the level of anxiety. All three outcomes failed to reveal a significant interaction effect between treatment conditions and time. Individuals undergoing cPPT did not display significantly different levels of positive outcomes over time compared to those in the mPPT.

Conversely, the main effect of time demonstrated statistical significance across all measures.

For the PPTI, there was no significant main effect of group ($F(1,102)=1.80,p=\mathrm{.549},\eta^{2}=0.01$), nor a significant interaction between group and time ($F(2,208)=0.57,p=\mathrm{.565},\eta^{2}=0.00,\eta_{w}^{2}=0.00$). However, the main effect of time was statistically significant ($F(2,208)=19.56,p<\mathrm{.001},\eta^{2}=0.04,\eta_{w}^{2}=0.15$). Time explained 4% of the total variance and 15% of the within-person variation in the PPTI. Pairwise comparisons showed that participants reported significantly higher levels of positive psychotherapy indicators at both post-treatment (diff=−7.28,t(208)=−5.96,p<.001) and follow-up (diff=−5.65,t(208)=−4.62,p<.001) compared to pre-treatment. In the FS scale, there was no significant main effect of group ($F(1,102)=0.57,p=\mathrm{.678},\eta^{2}=0.00$) and no significant interaction between group and time ($F(2,208)=0.80,p=\mathrm{.565},\eta^{2}=0.00,\eta_{w}^{2}=0.00$). A significant main effect of time was observed ($F(2,208)=8.02,p<\mathrm{.001},\eta^{2}=0.02,\eta_{w}^{2}=0.07$), accounting for 2% of the overall variance and 7% of the within-subject variance, in the FS scale. Pairwise comparisons revealed significantly greater flourishing scores at post-treatment (diff=−2.78,t(208)=−3.19,p=.005) and follow-up (diff=−3.23,t(208)=−3.69,p<.001) compared to pre-treatment. For the SWLS, neither the group effect ($F(1,102)=0.16,p=\mathrm{.693},\eta^{2}=0.00$) nor the interaction between group and time ($F(2,208)=1.37,p=\mathrm{.565},\eta^{2}=0.00,\eta_{w}^{2}=0.01$) was significant. However, a significant main effect of time emerged ($F(2,208)=17.84,p<\mathrm{.001},\eta^{2}=0.04,\eta_{w}^{2}=0.14$). In terms of variance, time contributed 4% to the total and 14% to the within-person component in SWLS. Pairwise comparisons showed significantly higher life satisfaction at both post-treatment (diff=−2.79,t(208)=−4.88,p<.001) and follow-up (diff=−3.10,t(208)=−5.42,p<.001) compared to pre-treatment.

**Fig 4:**
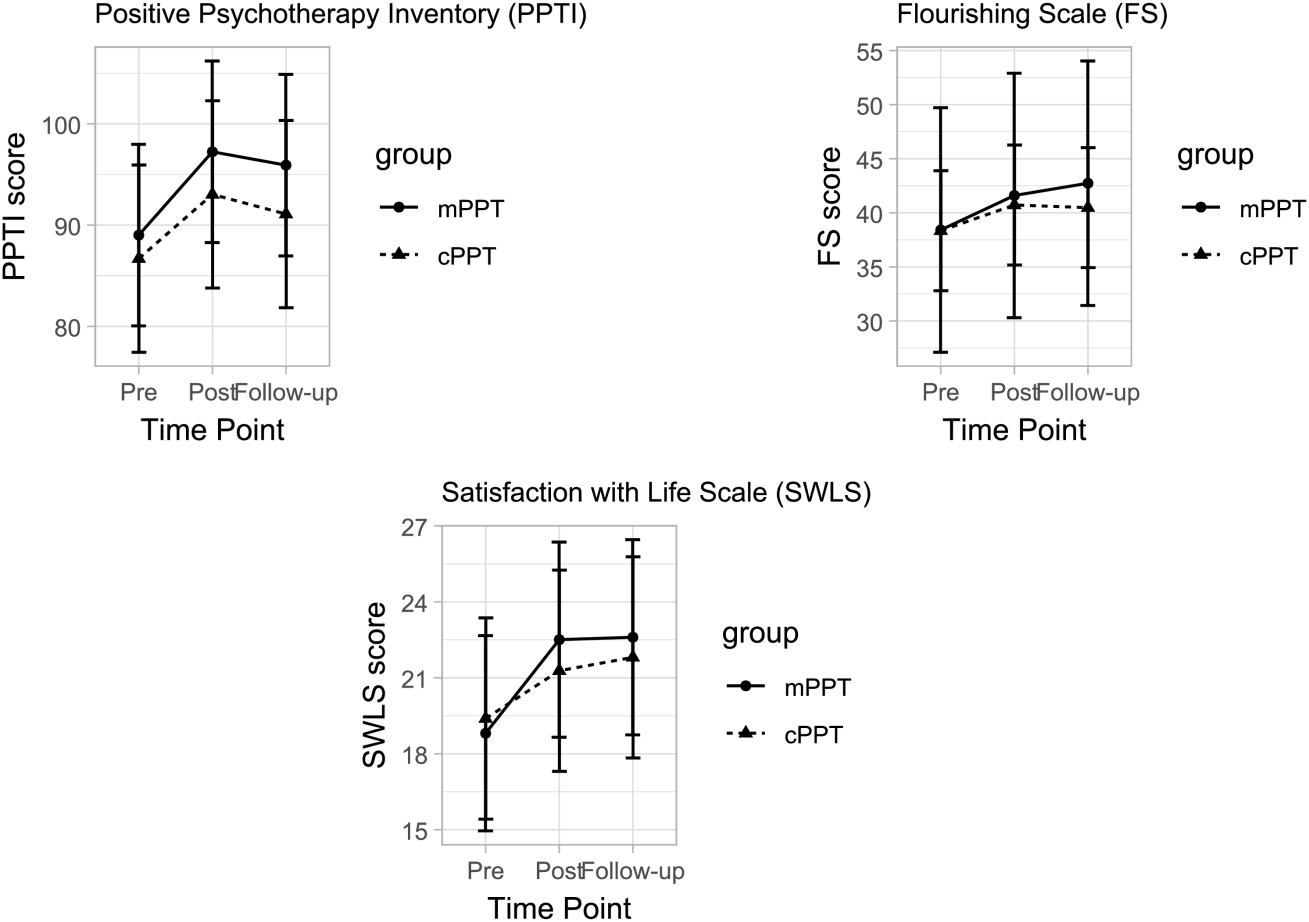
Plots of means for PPTI, FS, and SWLS.

Within both conditions, there are no statistically significant differences between post and follow-up measurements over time. Parallel patterns of findings are evident within the domains of flourishing and satisfaction. All reported results have been adjusted for the covariates of gender and age. Time effects across these outcomes showed small to medium effects, with $\eta^{2}$ ranging from .02 to .04 and $\eta_{w}^{2}$ ranging from .07 to .15. Comprehensive details of the results can be found in the corresponding Tables 5 and 6, and in Fig 4.

**Table 5:** *F*-tests for PPTI, FS, and SWLS.

	PPTI						FS						SWLS					
Factor	df1	df2	*F*	*p*	$\eta^{2}$	$\eta_{w}^{2}$	df1	df2	*F*	*p*	$\eta^{2}$	$\eta_{w}^{2}$	df1	df2	*F*	*p*	$\eta^{2}$	$\eta_{w}^{2}$
group	1	102.00	1.80	0.549	0.01	–	1	102.00	0.57	0.678	0.00	–	1	102.00	0.16	0.693	0.00	–
time	2	208.00	19.56	<0.001	0.04	0.15	2	208.00	8.02	<0.001	0.02	0.07	2	208.00	17.84	<0.001	0.04	0.14
group:time	2	208.00	0.57	0.565	0.00	0.00	2	208.00	0.80	0.565	0.00	0.00	2	208.00	1.37	0.565	0.00	0.01
gender	1	102.00	0.00	0.949	0.00	–	1	102.00	0.01	0.949	0.00	–	1	102.00	0.26	0.949	0.00	–
age	1	102.00	2.32	0.208	0.02	–	1	102.00	2.23	0.208	0.01	–	1	102.00	0.05	0.822	0.00	–

**Table 6:** Pairwise comparison for time for PPTI, FS, and SWLS.

	PPTI					FS					SWLS				
Contrast	Est	SE	df	*t*	*p*	Est	SE	df	*t*	*p*	Est	SE	df	*t*	*p*
pre-post	−7.28	1.22	208.00	−5.96	<0.001	−2.78	0.87	208.00	−3.19	0.005	−2.79	0.57	208.00	−4.88	<0.001
pre-(follow-up)	−5.65	1.22	208.00	−4.62	<0.001	−3.23	0.87	208.00	−3.69	<0.001	−3.10	0.57	208.00	−5.42	<0.001
post-(follow-up)	1.63	1.22	208.00	1.34	0.377	−0.44	0.87	208.00	−0.51	0.868	−0.31	0.57	208.00	−0.54	0.85

### Secondary outcomes

#### General psychological distress.

Examining indicators of general psychological distress via a linear mixed model reveals similar trends to earlier findings. A thorough analysis showed no significant interaction between treatment modality and time regarding ISR or PHQ9 scores. This indicates that individuals receiving cPPT did not experience notable changes in psychological distress levels over time compared to the mPPT. However, the main effect of time was statistically significant for both measures.

**Fig 5:**
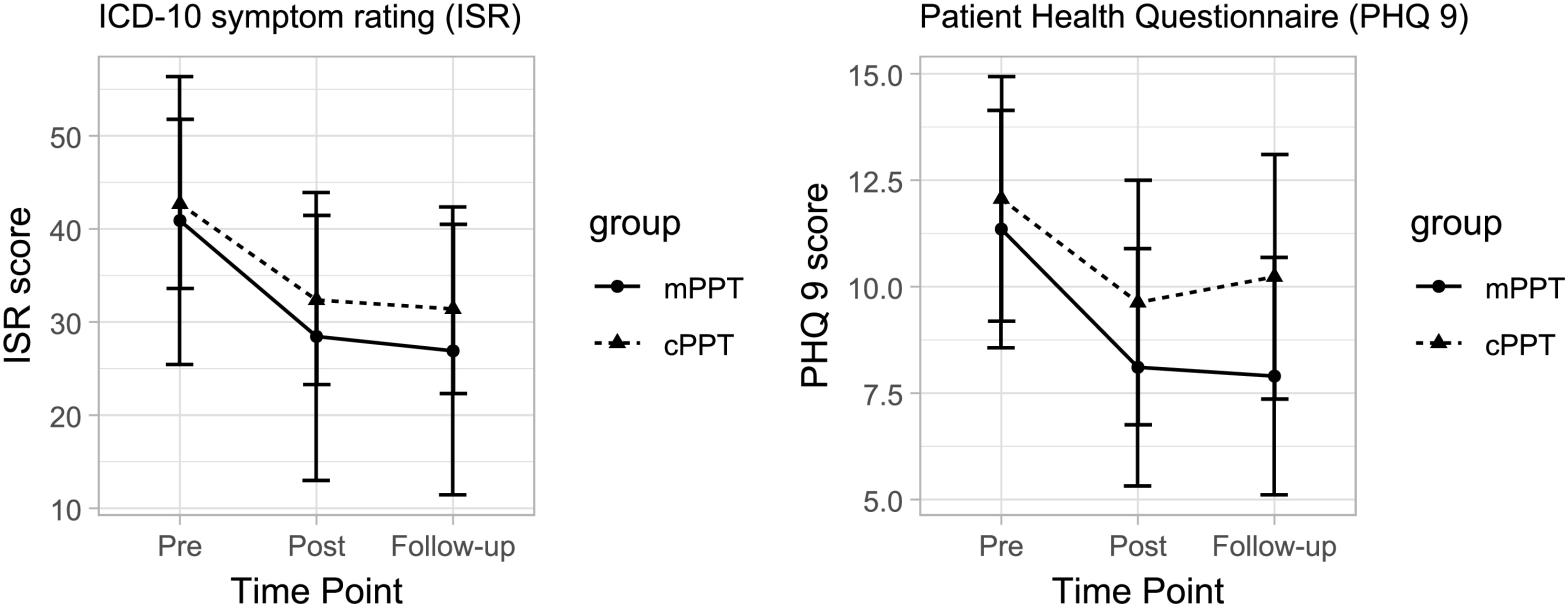
Plots of means for ISR and PHQ9.

In the ISR, there was no significant main effect of group ($F(1,102)=1.96,p=\mathrm{.165},\eta^{2}=0.01$), and the interaction between group and time was also non-significant ($F(2,208)=0.46,p=\mathrm{.629},\eta_{w}^{2}=0.00,\eta_{w}^{2}=0.00$). However, the main effect of time was statistically significant ($F(2,208)=44.09,p<\mathrm{.001},\eta^{2}=0.11,\eta_{w}^{2}=0.29$). The effect of time accounted for 11% of the total and 29% of the within-person variance in the ISR. Pairwise comparisons revealed that psychological distress scores (ISR) were significantly lower at post-treatment (diff=11.39,t(208)=7.68,p<.001) and follow-up (diff=12.64,t(208)=8.52,p<.001) compared to pre-treatment. For the PHQ-9, neither the group effect ($F(1,102)=3.09,p=\mathrm{.164},\eta^{2}=0.02$) nor the group × time interaction ($F(2,208)=1.45,p=\mathrm{.473},\eta^{2}=0.00,\eta_{w}^{2}=0.01$) was statistically significant. Nevertheless, the main effect of time was significant ($F(2,208)=22.19,p<\mathrm{.001},\eta^{2}=0.06,\eta_{w}^{2}=0.17$). A total of 6% between-person and 17% within-person variation was explained by time in the PHQ-9. Pairwise comparisons showed significantly lower depression scores at post-treatment (diff=2.84,t(208)=5.97,p<.001) and follow-up (diff=2.64,t(208)=5.55,p<.001) compared to pre-treatment. As in previous results, the general psychological distress variables showed almost parallel slope patterns in the graphs. Both groups exhibited a strong decline between pre- and post-measurement and an almost stable pattern between post and follow-up measurement. Again, all results have been adjusted for gender and age covariates. Time effects in these outcomes indicated small to medium effects in $\eta^{2}$, with $\eta^{2}$ ranging from .06 to .11 and large effects in $\eta_{w}^{2}$, ranging from .17 to .29. Comprehensive details of the results can be found in the corresponding Tables 7 and 8, and in Fig 5.

**Table 7:** *F*-tests for ISR and PHQ9.

	ISR						PHQ9					
Factor	df1	df2	*F*	*p*	$\eta^{2}$	$\eta_{w}^{2}$	df1	df2	*F*	*p*	$\eta^{2}$	$\eta_{w}^{2}$
group	1	102.00	1.96	0.165	0.01	–	1	102.00	3.09	0.164	0.02	–
time	2	208.00	44.09	<0.001	0.11	0.29	2	208.00	22.19	<0.001	0.06	0.17
group:time	2	208.00	0.46	0.629	0.00	0.00	2	208.00	1.45	0.473	0.00	0.01
gender	1	102.00	0.70	0.647	0.00	–	1	102.00	0.21	0.647	0.00	–
age	1	102.00	6.22	0.028	0.04	–	1	102.00	0.62	0.434	0.00	–

**Table 8:** Pairwise comparison for time ISR and PHQ9.

	ISR					PHQ9				
Contrast	Est	SE	df	*t*	*p*	Est	SE	df	*t*	*p*
pre-post	11.39	1.48	208.00	7.68	<0.001	2.84	0.48	208.00	5.97	<0.001
pre-(follow-up)	12.64	1.48	208.00	8.52	<0.001	2.64	0.48	208.00	5.55	<0.001
post-(follow-up)	1.25	1.48	208.00	0.85	0.675	−0.20	0.48	208.00	−0.42	0.909

## Discussion

This study aimed to compare the effectiveness of positive psychotherapy (PPT) delivered with full therapeutic support versus minimal therapeutic support in alleviating anxiety symptoms and overall psychological distress, and in improving happiness and quality of life among a large sample of German-speaking individuals with anxiety disorders. By creating a group with complete therapeutic support and a group with minimal therapeutic support, we investigated how the amount of therapist contact affected the outcome variables. The findings indicate a notable reduction in anxiety levels as well as significant improvements in feelings of happiness and quality of life throughout the therapy, maintaining consistency even three months post-treatment during the follow-up period in both groups. Similar outcomes were observed for the secondary measures, assessing the overall level of psychological distress, with lower scores post-intervention compared to pre-intervention. This trend persisted during the follow-up period compared to pre-intervention. While a successful therapy outcome was expected for the group with regular therapist support, the finding of all outcome variables improving similarly in the group with minimal therapeutic support throughout all measurement points was surprising. There is substantial evidence of therapy alliance being an important common factor for therapy success [42–44]. Studies, however, also showed that the importance of the quality of the therapeutic bond seems to depend in part on the client’s social support [45]. Thus, clients with less social support would benefit from a better therapist-patient relationship. In addition, social interaction is an important component of mental health, which is further impaired as the severity of the anxiety disorder increases. People who are socially isolated are at a higher risk of developing anxiety disorders [46]. Thus, the peer intervention in this study was implemented to additionally build a support system of instead people dealing with similar issues to socialize and share their struggles. This social contact may have also been an important aid to the therapy outcome. Results of several studies suggest that a therapist-patient relationship can also be accomplished through minimal contact with the therapist [47]. In a meta-analysis evaluating 31 studies, [48] compared regular face-to-face psychotherapy with therapist-supported Internet-based therapy. The results demonstrated no inferiority of guided self-help groups to face-to-face therapy, even leaning in favor of guided self-help groups. Similar findings were made in a meta-analysis by [49], with self-help treatments performing better than a waiting-list or placebo control group as well as face-to-face treatment-as-usual groups (i.e., psychotherapy without specific descriptions of treatment content). Therefore, albeit a good therapist-patient relationship seems to be of importance for therapy success, its intensity might not be as crucial. Another study compared the effectiveness of clinically guided internet-based cognitive behavioral therapy (ICBT) delivered in a group format, an individual format, and a waiting list for social anxiety disorder. The results showed that both active treatment conditions achieved better outcomes in terms of social anxiety symptoms compared to the waiting list. However, there were no significant differences between the two active conditions in terms of symptom reduction or treatment dropout. The treatment gains were maintained at follow-up. This indicates an efficiency of the ICBT group format, as the weekly therapist time was significantly reduced in the group format [50]. Given the substantial improvements observed in the minimal-support condition, blended PPT with reduced therapist contact may represent a promising and resource-efficient treatment option, particularly in contexts where access to therapist time is limited. By being administered online, therapy is accessible to a larger amount of people and could be more appealing to those who would not go into regular therapy otherwise. As anxiety disorders ultimately isolate sufferers from social contact [46] being in a group therapy can be a resourceful and easy way for people with anxiety disorders to resume a social life. Online therapy with minimal therapeutic support would be an affordable, highly economic, and efficient way to meet the demands of therapy if the intervention is designed correctly (e.g., regarding the material used, the amount and duration of the treatment sessions, and the availability of therapeutic support). Furthermore, this type of therapy could be a great way to bridge waitlist-time for individual and more therapist-guided therapy [51]. This study contributes to the progress of research by being, to the best of our knowledge, the most extensive randomized controlled trial with Positive Psychotherapy (in a blended therapy setting) for anxiety treatment, involving a sample of 106 participants. The results indicate that both levels of therapeutic support were associated with substantial and sustained improvements across outcomes, while no statistically significant differences between the two treatment conditions over time were detected. Importantly, although no statistically significant differences between the two treatment conditions were observed across time, this finding must not be interpreted as evidence of non-inferiority or equivalence of the minimal-support condition. The present study was not designed as a non-inferiority or equivalence trial, and no corresponding margins were defined.

### 5.1. Limitation

It is essential to recognize several limitations of this study. Firstly, with most participants being female (97.3%), the findings may not be broadly applicable to the overall population. Replicating the study with a more diverse sample is necessary to establish potential cross-gender effects. It should also be mentioned that due to the experienced drop-outs, a larger samples size should be considered that would better match our ambitious number of investigated primary outcomes. It should additionally be noted that the therapy was administered by nine different therapists, introducing complexity to comparisons. To mitigate therapist subjectivity, manual adherence was monitored through regular intervision and supervision. Both groups experienced subject loss and missing data, yet it is noteworthy that dropouts were evenly distributed between groups. Moreover, linear mixed models are resilient to missing data [52]. Since all participants were informed about the possibility of being allocated to the blended PPT group with minimal therapeutic support prior to study begin, the willingness and open-mindedness of the participants to try this condition might have been higher than usual. Since motivation and believe in treatment success are important factors in therapy, this might be a contributing factor to the more positive result of this study compared to others. Although the effectiveness of the blended PPT group with minimal therapeutic support regarding therapy outcome looks promising, it should be noted that this kind of therapy is not necessarily appropriate for all individuals. The severity of the disorder as well as comorbidity and suicidality are factors that should be considered carefully when choosing the treatment.

Additionally, it can be argued that the social aspect of this study (group setting, peer intervention) might have a significant impact on the therapy outcome, more so than the PPT itself. Thus, further studies should focus on a control group for that variable to bring more evidence to the efficacy of blended group PPT regardless of the amount of therapeutic support. Although the effectiveness of the blended PPT group with minimal therapeutic support appears promising, the absence of statistically significant differences between treatment conditions does not allow conclusions regarding non-inferiority or equivalence. Future studies using appropriate non-inferiority or equivalence designs are required to address this question explicitly.

### 5.2. Conclusion

Overall, the study demonstrates that blended group-based Positive Psychotherapy can lead to meaningful and lasting improvements in anxiety, psychological distress, happiness, and quality of life, even when therapist contact is substantially reduced. Participants in both groups showed significant improvements in all outcome measures, with the therapy goal being maintained up until the follow-up three months later. These findings suggest that, within the context of blended group-based Positive Psychotherapy, meaningful therapeutic improvements may be observed even when therapist contact is reduced. Further studies on the efficacy of blended therapy treatments, especially in the context of group PPT and in an online setting, are needed to strengthen the current evidence as well as assessing the therapist alliance to control for its effect on therapy outcome. Additionally, to gain more proof of the long-lasting effects of blended PPT, a follow-up later (e.g., one year) should be implemented. With further evidence, affordable and efficient alternatives to conventional therapy could be made in the form of minimal online (group) interventions to tackle anxiety disorders.

## Supporting information

S1 Protocol Study protocol German. Study protocol as approved by the ethics committee in original language (German).
(PDF)

S2 Protocol Study protocol English. English translation of the study protocol as approved by the ethics committee.
(PDF)

S3 Appendix CONSORT checklist. (PDF)

S4 Data Dataset. Raw data. Complete set of collected data collected during the study.
(CSV)
