# Supplementary material for: Extent of therapeutic support in Positive Psychotherapy: A randomized comparative efficacy study for the treatment of anxiety disorders in an online group setting
Source: PLoS One. 2026 Jul 28;21(7):e0354083. doi: 10.1371/journal.pone.0354083 (PMC13411940; doi:10.1371/journal.pone.0354083)
Supplement: S1 Protocol — Study protocol as approved by the ethics committee in original language (German). (PDF) [file pone.0354083.s001.pdf]

Antragsformular
zur Beurteilung eines Projekts durch die Ethikkommission der Universität Salzburg

Version 3.0 (Oktober 2019)

Raum für Eingangsstempel, EK-Nummer, etc. Bitte Freilassen!

Bitte die einzelnen Fragen mit den wesentlichen Informationen beantworten (i.d.R. max. 150 Worte, ausser bei 2.3). Nicht zutreffende Punkte bitte mit t.n.z. (trifft nicht zu) beantworten. Das Antragsformular ist in deutscher Sprache auszufüllen!

1. Allgemeines

1.1. Projekttitel

Online-Interventionsstudie bei Angststörungen mit Kognitiver Verhaltenstherapie und Positiver Psychologie

1.2. AntragstellerIn

Catiana Luisa Engelhardt, M.Sc.

1.3. Bei der vorgelegten Studie handelt es sich um:

- ☐ ein drittmittelfinanziertes Projekt

Universitätsinterne Projektnummer/Kostenstelle (falls vorhanden):

t.n.z.

- ☐ ein universitätsinternes Projekt
- ■ eine Dissertation (PhD-Arbeit)
- ☐ eine Masterarbeit/ Diplomarbeit

Name des Betreuers/der Betreuerin:

a.o. Univ.-Prof. Dr. Anton- Rupert Laireiter

Einreichung wurde verlangt von:

■ Betreuer/Betreuerin ☐ Studienrechtliches Organ

1.4. Warum wird ein Urteil der Ethikkommission benötigt (bei Publikation Angabe des Publikationsorgans)?

Es handelt sich um eine Studie zu einer Intervention mit Patienten*innen, die an einer Angststörung leiden (Generalisierte Angststörung, Soziale Angststörung/soziale Phobie und/oder Panikstörung mit oder ohne Agoraphobie). Die Publikation dreier Paper ist geplant (Publikationsorgan noch nicht bekannt)

1.5. Hat das Projekt bereits ein Begutachtungsverfahren durchlaufen?

☐ Ja ■ Nein ☐ Wenn ja, bei welcher Institution? Gutachten beilegen!

1.6. Liegen bereits Voten anderer Ethikkommissionen vor?

☐ Ja ■ Nein ☐ Wenn ja, Voten beilegen!

2. Strukturierte Kurzfassung des Projekts

2.1. Art des Projekts

Es handelt sich um eine Online-Interventionsstudie im Gruppensetting mit einer Wirksamkeits- und einer Prozessstudie. Verglichen werden die Kognitive Verhaltenstherapie und die Positive Psychotherapie

2.2. Fachgebiet

Klinische Psychologie, Psychotherapie, Positive Psychologie

2.3. Allgemeine Kurzzusammenfassung des Projekts (Hintergrund, Fragestellungen, Methoden, max. 500 Wörter)

In der geplanten Untersuchung soll eine randomisierte kontrollierte Vergleichsstudie an Angstpatienten*innen mit Generalisierter Angststörung, sozialer

Angststörung/sozialer Phobie und/oder Panikstörung mit oder ohne Agoraphobie mit zwei aktiven Behandlungsbedingungen (Positive Psychotherapie und Kognitive Verhaltenstherapie) und einer Vergleichsgruppe (Online Positive Psychotherapie mit minimaler therapeutischer Begleitung) im Gruppensetting im Online-Format (Telegruppen) durchgeführt werden. Inkludiert in diese Studie ist weiters eine Prozess-Wirksamkeitsstudie zur Ermittlung effektmoderierender (wirksamer) Faktoren.Die aus der Positiven Psychologie entstandene Positive Psychotherapie hat sich zum Ziel gesetzt einen positiven Fokus in die psychotherapeutische Arbeit einzubringen. Für die Positive Psychotherapie wurde ein neues Behandlungsmanual, welches sich an das von Rashid und Seligman (2018) anlehnt, entwickelt. Für die Kognitive Verhaltenstherapie wurde ebenfalls ein neues - übergeordnetes (auf alle drei Angststörungen anwendbares) - Behandlungsmanual in Anlehnung an das „Therapietool Angststörungen" von Hagena und Gebauer (2014) konzipiert. Das Behandlungsmanual der Kontrollgruppe ist thematisch an das der Positiven Psychotherapie angelehnt, jedoch stark gekürzt. Die Behandlungsmanuale der PPT und der KVT umfassen jeweils 12 Gruppensitzungen á 90-100 Minuten und eine Einzelstunde. Das Behandlungsmanual der Kontrollgruppe umfasst 12 Sitzungen, von denen drei (die erste, die sechste und die zwölfte) im Online-Gruppensetting stattfinden und die restlichen neun mithilfe von Lehrvideos und E-Mail Verkehr selbstständig erarbeitet werden. Alle Therapiestunden finden online statt. Die einzelnen Gruppen bestehen aus sechs bis acht Teilnehmer*innen. Die Teilnehmer*innen jeder Gruppe er- halten zudem Zugriff auf eine eigens für die Studie angelegte Website. Auf dieser Website stehen Arbeitsmaterialien, Audiodateien und Lehrvideos zur Verfügung. Zudem bietet sie die Möglichkeit, über eine Chatfunktion Kontakt mit der Studienleitung aufzunehmen; auch dient sie als virtuelle Pinnwand, um bestimmte Übungen in anonymisierter Form sichtbar zu machen. Die Studie umfasst drei Messzeit- punkte, eine Prä- und Post-Messung und ein Follow-up nach drei Monaten. Die Behandlungen werden von Psychologen*innen in Ausbildung zur/m Klinischen Psycholog*in und Psychologische/n Psychotherapeuten*in in Österreich und Deutschland durchgeführt. Alle Psycholog*innen werden intensiv in die jeweiligen Manuale eingeschult, nehmen an regelmäßigen Intervisionssitzungen teil und erhalten in regelmäßigen Abständen Supervision von erfahrenen Vertreter*innen der Positiven Psychologie und der Kognitiven Verhaltenstherapie. Alle Gruppensitzungen werden protokolliert und in der Supervision nachbesprochen. Jede Sitzung wird auf ihre Manualtreue (Adhärenz) hin überprüft. Wie eingangs bereits gesagt ist in die Studie auch eine Prozess-Wirksamkeits- Studie inkludiert. In dieser sollen sowohl die Intra-Session-Prozesse als auch die Intersession-Prozesse der Therapie mit Positiver Psychotherapie und Kognitiver Verhaltenstherapie evaluiert und mit dem Therapie-Outcome in Beziehung gesetzt werden. Unter Intra-Session-Prozessen werden all jene Veränderungen, die unmittelbar als Reaktion auf bestimmt Ereignisse in der Therapiesitzung auftreten, verstanden, während die Intersession-Prozesse als Veränderungsprozesse zwischen zwei Sitzungen, die durch Erinnerungen und Repräsentationen der Sitzung her- vorgerufen werden, definiert sind (Orlinsky & Geller, 1993). Die Intra-Session- Prozesse sollen mit Hilfe des Berner Stundenbeurteilungsbogen für Patienten (Flückiger et al., 2010) erfasst werden, wobei von den Patient*innen nach jeder Sitzung erfasst wird, sie diese erlebt haben und ob diese aus ihrer Sicht einen Fortschritt gebracht hat, zum einem im Sinne des

Verständnisses für die Probleme und Einsicht in die Zusammenhänge, zum anderen im Sinne des Eindrucks, die eigenen Probleme fortan besser bewältigen zu können. Die Intersession-Prozesse werden mit Hilfe des Intersession-Fragebogens Hartmann und Orlinsky erfasst. Neben der Erfassung der Prozesse soll insbesondere untersucht werden, inwiefern sich die Positive Psychotherapie und die Kognitive Verhaltenstherapie in den Therapieprozessen (Intra- und Intersession-Prozesse) unterscheiden und welche Variablen beider Prozesstypen den Therapieerfolg voraussagen. Die Kontrollgruppe ist in die Prozessstudie nicht einbezogen. Sie dient ausschließlich als Vergleichsgruppe der Wirksamkeitsstudie, um zu überprüfen, welchen Effekt eine niederschwellig angebotene Positive Psychologie-Intervention im Vergleich zu einer angeleiteten erbringt. Um den Effekt der Prozessvariablen auf das Therapieergebnis (Rückgang der Angstsymptomatik und der allgemeinen psychischen Belastetheit, Anstieg des Glückserlebens, der Lebenszufriedenheit und der Lebensqualität) zu überprüfen, werden zwei globale Indikatoren gebildet: 1. Häufigkeit und Intensität der Intra- und Intersession-Aktivitäten und 2. die emotionalen Valenz der Intra- und Interession-Prozesse (positiv vs. negativ). Ziel dieser Studie ist es, Veränderungsvariablen zu identifizieren, Erkenntnisse über die Therapievorgänge zu gewinnen und somit den Therapieprozess zu optimieren.

2.4. Zielsetzung, Fragestellungen, Hypothesen (ggf.)

In der Effektivitätsstudie soll geklärt werden, inwieweit die Positive Psychotherapie im Vergleich zur kognitiven Verhaltenstherapie und einer Kontrollgruppe (Online Positive Psychotherapie mit minimaler therapeutischer Begleitung - im Folgenden der Kürze wegen nur noch als "minimal begleitete Online PPT" genannt) bei Patienten*innen mit einer Generalisierten Angststörung, einer sozialen Angststörung/sozialen Phobie und/oder einer Panikstörung mit oder ohne Agoraphobie (im Folgenden der Kürze wegen nur noch "Angstpatient*innen") die Angstsymptomatik reduzieren und die allgemeine Lebenszufriedenheit, die Lebensqualität und das Glückserleben erhöhen kann. Durch diese Studie soll die Wirksamkeit der Positiven Psychotherapie bei der Behandlung von Angststörungen erstmals auch im Online-Format evaluiert werden.

Forschungshypothesen:

1) Die Angstpatienten*innen, die an dem Behandlungsprogramm der Positiven Psychotherapie teilgenommen haben, zeigen einen signifikanten Rückgang der Angstsymptomatik zwischen Prä- und Postmessung.
2) Die Angstpatienten*innen, die an dem Behandlungsprogramm der Positiven Psychotherapie teilgenommen haben, zeigen einen signifikant stärkeren Rückgang der Angstsymptomatik im Vergleich zu den Patient*innen, die an dem Behandlungsprogramm der minimal begleiteten Online PPT teilgenommen haben.
3) Die Angstpatienten*innen, die an dem Behandlungsprogramm der Kognitiven Verhaltenstherapie teilgenommen haben, zeigen einen signifikanten Rückgang der Angstsymptomatik zwischen Prä- und Postmessung.

4) Die Angstpatienten*innen, die am Behandlungsprogramm der Kognitiven Verhaltenstherapie teilgenommen haben, zeigen einen signifikant stärkeren Rückgang der Angstsymptomatik im Vergleich zu den Patient*innen, die an dem Behandlungsprogramm der minimal begleiteten Online PPT teilgenommen haben.
5) Die Angstpatienten*innen, die an dem Behandlungsprogramm der Positiven Psychotherapie teilgenommen haben, zeigen einen vergleichbaren Rückgang der Angstsymptomatik, wie die Patient*innen, die an dem Behandlungsprogramm der Kognitiven Verhaltenstherapie teilgenommen haben.
6) Die Angstpatienten*innen, die an dem Behandlungsprogramm der Positiven Psychotherapie teilgenommen haben, erleben durch direkte Einflussnahme auf positive Emotionen, auf das Erkennen und den Gebrauch der eigenen Stärken sowie auf das Erleben von Sinn einen signifikanten Anstieg im Glückserleben zwischen Prä- und Postmessung.
7) Die Angstpatienten*innen, die an dem Behandlungsprogramm der Positiven Psychotherapie teilgenommen haben, erleben durch direkte Einflussnahme auf positive Emotionen, auf das Erkennen und den Gebrauch der eigenen Stärken sowie auf das Erleben von Sinn einen signifikant stärkeren Anstieg im Glückserleben im Vergleich zu den Patient*innen, die an der minimal begleiteten Online PPT teilgenommen haben.
8) Die Angstpatienten*innen, die an dem Behandlungsprogramm der Positiven Psychotherapie teilgenommen haben, erleben durch direkte Einflussnahme auf positive Emotionen, auf das Erkennen und den Gebrauch der eigenen Stärken sowie auf das Erleben von Sinn einen signifikant stärkeren Anstieg im Glückserleben im Vergleich zu den Patient*innen, die an dem Behandlungsprogramm der Kognitiven Verhaltenstherapie teilgenommen haben.
9) Die Angstpatienten*innen, die an dem Behandlungsprogramm der Positiven Psychotherapie teilgenommen haben, erleben durch direkte Einflussnahme auf positive Emotionen, auf das Erkennen und den Gebrauch der eigenen Stärken sowie auf das Erleben von Sinn einen signifikanten Anstieg hinsichtlich der Lebenszufriedenheit und Lebensqualität zwischen Prä- und Postmessung.
10) Die Angstpatienten*innen, die an dem Behandlungsprogramm der Positiven Psychotherapie teilgenommen haben, erleben durch direkte Einflussnahme auf positive Emotionen, auf das Erkennen und den Gebrauch der eigenen Stärken sowie auf das Erleben von Sinn einen signifikant stärkeren Anstieg hinsichtlich der Lebenszufriedenheit und Lebensqualität als die Patient*innen, die an dem Behandlungsprogramm der minimal begleiteten Online PPT teilgenommen haben.
11) Die Angstpatienten*innen, die an dem Behandlungsprogramm der Positiven Psychotherapie teilgenommen haben, erleben durch direkte Einflussnahme auf positive Emotionen und das Selbstmitgefühl, auf das Erkennen und den Gebrauch der eigenen Stärken, sowie auf das Erleben von Sinn einen signifikant stärkeren Anstieg in der Lebenszufriedenheit und Lebensqualität als die Patient*innen, die an dem Behandlungsprogramm der Kognitiven Verhaltenstherapie teilgenommen haben.

12) Der signifikanten Rückgang der Angstsymptomatik bei den Patienten*innen, die an dem Behandlungsprogramm der Positiven Psychotherapie teilgenommen haben, bleibt auch über einen Zeitraum von drei Monaten stabil.
13) Der signifikanten Rückgang der Angstsymptomatik bei den Patienten*innen, die an dem Behandlungsprogramm der Kognitiven Verhaltenstherapie teilgenommen haben, bleibt auch über einen Zeitraum von drei Monaten stabil.
14) Der signifikanten Anstieg im Glückserleben bei den Patienten*innen, die an dem Behandlungsprogramm der Positiven Psychotherapie teilgenommen haben, bleibt auch über einen Zeitraum von drei Monaten stabil.
15) Der signifikanten Anstieg hinsichtlich der Lebenszufriedenheit und Lebensqualität bei den Patienten*innen, die an dem Behandlungsprogramm der Positiven Psychotherapie teilgenommen haben, bleibt auch über einen Zeitraum von drei Monaten stabil.
16) Im Sinne einer explorativen Fragestellung wird davon ausgegangen, dass die Effekte der Online Positiven Psychotherapie mit minimaler therapeutischen Begleitung über drei Monate nicht stabil bleiben werden.

Im Rahmen der Prozess-Studie soll der Prozess der Therapie in Positiver Psychotherapie und Kognitiver Verhaltenstherapie evaluiert werden. In der Prozess-Studie sollen sowohl die Intra-Session-Prozesse als auch die Intersession-Prozesse der Therapie mit Positiver Psychotherapie und Kognitiver Verhaltenstherapie evaluiert werden. Des Weiteren soll erfasst werden, welche Prozessvariablen (Intra-Session- oder Intersession-Prozesse) den Therapieerfolg voraussagen und inwiefern sich die Positive Psychotherapie und die Kognitive Verhaltenstherapie in den Therapiepro- zessen unterscheiden. Die Intra-Session-Prozesse sollen mit Hilfe des Stundenbeurteilungsbogen für Patienten (STU-P) erfasst werden. Die Teilnehmer*innen füllen diesen Fragebogen nach jeder Sitzung in digitaler Form aus. Die Intersession- Prozesse sollen mit Hilfe des Intersession-Fragebogens (ISF) erfasst werden. Diesen füllen die Teilnehmer*innen vor jeder Sitzung in digitaler Form aus. Ziel ist es, den Prozess zu untersuchen und zu bewerten. Eventuell können so auch noch nach Beendigung der Studie Änderungen an den Manualen vorgenommen und Erkenntnisse über die Therapievorgänge gewonnen werden. Ziel ist es, therapeutische Prozessvariablen zu identifizieren und somit den Therapieprozess zu optimieren.

Forschungshypothesen:
1) Angstpatienten*innen, deren Intersession-Aktivität emotional positiv besetzt ist, weisen einen stärkeren Rückgang ihrer Angstsymptomatik auf als Patient*innen, deren Intersession-Aktivität negativ besetzt ist. Es besteht ein signifikanter Zusammenhang zwischen der emotionalen Besetzung der Intersession- Prozesse und dem Rückgang der Angstsymptomatik.
2) Angstpatienten*innen, deren Intersession-Aktivität emotional positiv besetzt ist, weisen einen stärkeren Anstieg ihres Glückserlebens auf als Patient*innen, deren Intersession-Aktivität negativ besetzt ist. Es besteht ein signifikanter Zusammenhang zwischen der emotionalen Besetzung der Intersession-Prozesse und dem Anstieg des Glückserlebens.

3) Angstpatienten*innen, deren Intersession-Aktivität emotional positiv besetzt ist, weisen einen stärkeren Anstieg in ihrer Lebenszufriedenheit und ihrer Lebensqualität auf als Patient*innen, deren Intersession-Aktivität negativ besetzt ist. Es besteht ein signifikanter Zusammenhang zwischen der emotionalen Besetzung der Intersession-Prozesse und dem Anstieg der Lebenszufriedenheit und der Lebensqualität.
4) Angstpatienten*innen, deren Intra-Session-Aktivität emotional positiv besetzt ist, weisen einen stärkeren Rückgang ihrer Angstsymptomatik auf als Patient*innen, deren Intra-Session-Aktivität negativ besetzt ist. Es besteht ein signifikanter Zusammenhang zwischen der emotionalen Besetzung der Intra-Session- Prozesse und dem Rückgang in der Angstsymptomatik.
5) Angstpatienten*innen, deren Intra-Session-Aktivität emotional positiv besetzt ist, weisen einen stärkeren Anstieg im Glückserlebens auf als Patient*innen, deren Intra-Session-Aktivität negativ besetzt ist. Es besteht ein signifikanter Zusammenhang zwischen der emotionalen Besetzung der Intra-Session-Prozesse und dem Anstieg des Glückserlebens.
6) Angstpatienten*innen, deren Intra-Session-Aktivität emotional positiv besetzt ist, weisen einen höheren Anstieg in ihrer Lebenszufriedenheit und ihrer Lebensqualität auf als Patient*innen, deren Intra-Session-Aktivität negativ besetzt ist. Es besteht ein signifikanter Zusammenhang zwischen der emotionalen Besetzung der Intra-Session-Prozesse und dem Anstieg der Lebenszufriedenheit und der Lebensqualität.
7) Angstpatienten*innen, die ein höheres Ausmaß an Intersession-Aktivität aufweisen, zeigen am Ende der Therapie einen stärkeren Rückgang in ihrer Angstsymptomatik, als jene, die eine geringere Intersession-Aktivität aufweisen. Es besteht ein signifikant positiver Zusammenhang zwischen der Intensität und der Häufigkeit von Intersession-Aktivitäten und dem Rückgang der Angstsymptomatik.
8) Angstpatienten*innen, die ein höheres Maß an Intersession-Aktivität aufweisen, zeigen am Ende der Therapie einen stärkeren Anstieg ihres Glückserlebens, als je-ne, die weniger Intersession-Aktivität aufweisen. Es besteht ein signifikant positiver Zusammenhang zwischen der Intensität und der Häufigkeit von Intersession-Prozessen und dem Anstieg des Glückserlebens.
9) Angstpatienten*innen, die ein höheres Maß an Intersession-Aktivität aufweisen, zeigen am Ende der Therapie einen stärkeren Anstieg in ihrer Lebenszufriedenheit und ihrer Lebensqualität, als jene, die weniger Intersession-Aktivität aufweisen. Es besteht ein signifikant positiver Zusammenhang zwischen der Intensität und der Häufigkeit von Intersession-Prozessen und dem Anstieg an Lebenszufriedenheit und Lebensqualität.
10) Angstpatienten*innen, die ein höheres Maß an Intra-Session-Aktivität aufweisen, zeigen am Ende der Therapie einen stärkeren Rückgang der Angstsymptomatik, als jene, die weniger Intra-Session-Aktivität aufweisen. Es besteht ein signifikant positiver Zusammenhang zwischen der Intensität und der Häufigkeit von Intra-Session-Prozessen und dem Rückgang der Angstsymptomatik.
11) Angstpatienten*innen, die ein höheres Maß an Intra-Session-Aktivität aufweisen, zeigen am Ende der Therapie einen stärkeren Anstieg des Glückserle-

bens, als jene, die weniger Intra- Session-Aktivitäten aufweisen. Es besteht ein signifikant positiver Zusammenhang zwischen der Intensität und Häufigkeit an Intra-Session-Prozesse und dem Anstieg des Glückserlebens.

12) Angstpatienten*innen, die ein höheres Maß an Intra-Session-Aktivität aufweisen, zeigen am Ende der Therapie einen stärkeren Anstieg an Lebenszufriedenheit und Lebensqualität, als jene, die weniger Intra-Session-Aktivität aufweisen. Es besteht ein signifikant positiver Zusammenhang zwischen der Intensität und der Häufigkeit von Intra-Session-Prozessen und dem Anstieg an Lebenszufriedenheit und Lebensqualität.

2.5. Wissenschaftliche und gesellschaftliche Relevanz (Begründung des Projekts)

Die wissenschaftliche Relevanz dieser Studie ist sehr hoch. Es gibt aktuell nur eine geringe Anzahl an Studien, die die Effektivität der Positiven Psychotherapie bei klinischen Patienten*innen untersucht hat. Die meisten der vorhandenen Studien wurden an Patienten*innen mit depressiver Störung durchgeführt. Hier konnte eine hohe Wirksamkeit der Behandlung mit Positiver Psychotherapie festgestellt werden (z.B. Laireiter & Furchtlehner, 2018). Die angestrebte Untersuchung wäre die erste, die die Effektivität der Verfahren Kognitive Verhaltenstherapie und Positive Psychotherapie bei Angsstörungen im Gruppensetting unter Online-Bedingungen untersucht und vergleichend evaluiert. Die geplante Prozessstudie ist nach dem Wissen der Antragstellerin überhaupt die erste, die zu diesem Thema im Kontext der Positiven Psychotherapie jemals durchgeführt worden ist. Ihre Ergebnisse können daher wichtige und richtungsweisende Ergebnisse im Hinblick auf therapeutische Prozesse und Veränderungen in der Positiven Psychologie erbringen. Auch die gesellschaftliche Relevanz ist als sehr hoch einzuschätzen. Angststörungen sind die häufigsten psychischen Störungen überhaupt (Jacobi et al., 2004; Kessler et al., 2005). Die Lebenszeitprävalenz liegt internationalen Studien zufolge zwischen 14 und 29 % (Kessler et al., 2005; Somers et al., 2006). Die Lebensqualität der Betroffenen ist durch ihre Störung sehr stark eingeschränkt. Hinsichtlich der sogenannten „Years Lived with Disability“ (YLD), also der mit Behinderung gelebten Lebensjahre, lagen Angststörungen laut der Weltgesundheitsorganisation (WHO) im Jahr 2015 weltweit auf dem sechsten, in den hoch entwickelten (Industrie)Ländern sogar auf dem vierten Rang innerhalb aller psychischen und somatischen Störungen. Die Positive Psychotherapie stellt im Vergleich zur bisherigen, eher defizitorientierten Psychotherapie einen sehr vielversprechenden, innovativen Ansatz dar, nicht nur die Symptomatik zu reduzieren, sondern vor allem auch das Wohlbefinden, die Lebensqualität und die Lebenszufriedenheit zu fördern.

2.6.Forschungsdesign

3x3 varianzanalytisches Within-Between Subjects-Design (within-Subjects: Messwiederholungsfaktor mit drei Messzeitpunkten: Prä, Post, Follow-up nach drei Monaten). Between Subjects: Drei Behandlungsgruppen: zwei online

durchgeführte Interventions- und eine low-level guided Vergleichsgruppe Kontrollierte Interventionsstudie auf der Basis von verschriftlichten Behandlungsmanualen; explizierte Ein- und Ausschlusskriterien (s.u.); rationale Stichprobenplanung (s.u.); randomisierte Patient*innenzuweisung => RCT.

2.7. Beschreibung des Untersuchungsdesigns (Erhebungszeitpunkte, Art und Anzahl der Gruppen, Kontrollgruppen usw.)

In den folgenden Ausführungen werden die verwendeten Verfahren mit den gängigen Abkürzungen dargestellt; eine präzisiere Beschreibung findet sich weiter unten. Die Studie umfasst insgesamt fünf Messzeitpunkte, zwei Screening- (Vorauswahl und telefonisches/ videogestütztes Gespräch) und drei therapiebezogene Messungen.

1. Online Vorauswahl zur Prüfung der generellen Eignung mittels GAD-7, BAI, FQ und PAS und demographischen und klinischen Variablen

2. Das zweite Screening findet in Form eines Telefon- oder Videogesprächs vor Beginn der Intervention statt. Hier werden die Ausschlusskritieren mittels Mini-DIPS und PSS-K überprüft. Zudem werden alle Patienten*innen über den Ablauf der Studie und die Inhalte umfassend aufgeklärt und ihre explizite Zustimmung zur Teilnahme an der Studie nach den Studienkriterien wird eingeholt. Alle Teilnehmer*innen erhalten zudem die Rufnummer für den, für ihr Gebiet zuständigen, Krisendienst, um sich bei Notfällen an diese wenden zu können.

4. Die in die Studie aufgenommenen Teilnehmer*innen, die den Ein- und Ausschlusskriterien entsprechen, werden in eine der drei Interventionsgruppen (PPT, KVT, minimal begleitete PPT) aufgenommen. Die Zuteilung erfolgt randomisiert. Daran anschließend erhalten sie einen Link zu dem Onlinefragebogen und füllen online die Fragebögen zur Erfassung der abhängigen Baseline-Variablen aus (T1). Es werden folgende Selbstbeurteilungsfragebögen die Angst betreffend vorgegeben: Beck Anxiety Inventory (BAI), Panik und Agoraphobie Skala (PAS), Generalized Anxiety Disorder 7 (GAD-7), Fragebogen zur Sozialen Angst und sozialen Kompetenzdefiziten (SASKO), Angstfragebogen (AF)/Fear Questionnaire (FQ), Gesundheitsfragebogen für Patienten (PHQ-9), ICD-10- Symptom Rating (ISR) zur Erfassung der allgemeine psychischen Auffälligkeit. Positive Outcomes: Positives Psychotherapie-Inventar (PPTI), Flourishing- Scale (FS), Satisfaction with Life-Scale (SWLS); Fremdbeurteilung: Auf der Basis des klinischen Interviews werden seitens des/ der Interviewers*in folgende Verfahren bearbeitet: MiniDIPS (psychische Störungen), PSS-K (Persönlichkeitsstörungen), SCID PD (Persönlichkeitsstörungen) Panik und Agoraphobie-Skala (PAS) und Hamilton-Angst-Skala (HAMA).

5. Die 12- wöchige Intervention basierend auf den beiden Manualen wird durch Psychologen*innen in Ausbildung zur/m Klinischen Psychologen*in bzw. Psychologische/n Psychotherapeuten*in im dual Trainer-Setting (zwei Therapeut*innen) durchgeführt. Alle Therapeuten*innen werden intensiv in

den jeweiligen Manualen geschult, intervidieren ihre Arbeit regelmäßig und erhalten zusätzlich in regelmäßigen Abständen Supervision von erfahrenen Vertreter*innen der Positiven Psychologie und der Kognitiven Verhaltenstherapie. Alle Gruppensitzungen werden protokolliert und in der Supervision nachbesprochen. Jede Sitzung wird auf ihre Manualtreue hin beurteilt.
6. Vor jeder Therapieeinheit füllen die Teilnehmer*innen zur Prozessanalyse der Therapien den Intersession-Fragebogen (ISF) und nach jeder Einheit den Stundenbeurteilungsbogen für Patienten (STU-P) online aus.
7. Die zweite Fragebogenerhebung (T2) findet nach der letzten Therapieeinheit statt. Dazu erhalten die Teilnehmer*innen den Link zur Onlinebefragung erneut zugeschickt. Es werden die gleichen Selbstbeurteilungsverfahren wie zu T1 vorgegeben. Auch dieses Mal findet wiederum eine Fremdbeurteilung statt. Diese erfolgt mittels Fremdbeurteilungsskala der Panik und Agoraphobie- Skala (PAS) und der Hamilton-Angst-Skala (HAMA) durch die Therapeut*innen.
8. Drei Monate nach Beendigung der Online-Gruppen erhalten die Teilnehmer*innen erneut eine Email samt Link und dem Ersuchen um Bearbeitung der Fragebogenbatterie zugeschickt (=Follow-Up, T3). Auch dieses Mal werden die gleichen Selbstbeurteilungsverfahren wie zu T1 und T2 vorgegeben. Zu diesem Messzeitpunkt findet keine Fremdbeurteilung mehr statt.

2.8. Beschreibung der Methoden der Datenerhebung, Instrumente

In die Datenerhebung sind, wie aus vorangegangenem Abschnitt hervorgeht, sowohl Selbst- als auch Fremdbeurteilungsverfahren integriert. In beiden Fällen werden psychometrisch geprüfte klinische Interviews und Skalen verwendet. Die erfassten Merkmale der Wirksamkeitsstudie werden in primäre und sekundäre Outcomes unterteilt und umfassen sowohl die Angstausprägung wie auch zusätzliche psychopathologische Merkmale.

Personbezogene und soziodemographische Daten:
- Name, Wohnort, E-Mailadresse, Telefonnummer; Alter, Geschlecht, Familienstand, Bildung, Nationalität

Screening und Diagnostik:
- MiniDIPS: (aktuelle und Life-time)Diagnosen psychischer Störungen
- PSS-K: Persönlichkeitsstörungs-Screening-Kurzform
- SCID PD: Strukturiertes Klinisches Interview-Persönlichkeitsstörungen (bei Hinweisen nach PSS-K)
- Weitere Klinische Daten: psychometrische Daten aus den klinischen Verfahren, psychiatrische Behandlung, Einnahme von Psychopharmaka, vergangene Psychotherapie/n (Anzahl, Art)

Primäre Outcomes:
1. Angst Symptomatik:

Selbstbeurteilung	BAI, PAS; GAD-7, FQ
Fremdbeurteilung	PAS, HAMA
2. Positive Outcome	
Positive Psychotherapie-Inventar	PPTI
Flourishing Scale	FS
Satisfaction with Life-Scale	SWLS
Sekundäre Outcome:	
ICD-10-Symptom-Rating	ISR
Gesundheitsfragebogen für Patienten	PHQ-9
Prozessstudie:	
Stundenbeurteilungsbogen für Patienten	STU-P
Intersession-Fragebogen	ISF

2.9.Beschreibung der Stichprobe (TeilnehmerInnen)

Die Stichprobengröße wurde mittels einer Power Analyse (G*Power) errechnet. Diese beläuft sich auf 165 Personen; entsprechend würden 55 Personen in jede der drei Interventionsgruppen aufgenommen werden. Unabhängig davon werden für die Studie folgende Ein- und Ausschlusskriterien festgesetzt:

Einschlusskriterien:

- Alter von 18 bis 65
- Diagnose einer der folgenden Angststörungen: F40.1: Soziale Angststörung/soziale Phobie; F41.0 Panikstörung mit oder ohne Agoraphobie, F41.1 Generalisierte Angststörung
- ausreichende Deutschkenntnisse
- durchschnittliche intellektuelle Leistungsfähigkeit (Einschätzung während des telefonischen/videogestützen Screenings)
- ausreichende Zeit für die wöchentlichen Einheiten und Hausaufgaben
- Akzeptanz des Studienprotokolls

Ausschlusskriterien:

- eine gleichzeitig oder in den folgenden drei Monaten geplante Teilnahme an einer psychotherapeutischen und/oder psychologischen Behandlung oder Beratung sowie eine Teilnahme an sonstigen psychologischen Gruppenangeboten
- Symptome einer schweren Depression, einer bipolaren affektiven Störung oder Manie (aktuell sowie anamnestisch), psychotische, schizophrene und/

oder schizoaffektive Störungen, akute Trauerreaktion, schwere Anorexie oder Bulimie, Substanzabhängigkeiten (Alkohol, illegale Drogen)
- schwere Persönlichkeitsstörungen, insbesondere Borderline-, narzißtische, anti-/dissoziale und paranoide Persönlichkeitsstörung
- akute Suizidalität
- Umstellung, Dosisveränderung oder das vollständige Absetzen von Psychopharmaka in den vergangenen oder folgenden drei Monaten
- Fehlende Entscheidungsfähigkeit

2.10. Beschreibung der Erhebungsorte

Sowohl das Screening als auch die Intervention und die Erhebungen finden im Online-Format statt. Für die Durchführung der Online-Interventionen werden jedoch für die Psychologen*innen fixe Standorte festgelegt. In Salzburg werden die Online-Interventionen in den Räumlichkeiten der Beratungsstelle für Klinische Psychologie, Psychotherapie & Gesundheitspsychologie durchgeführt.

2.11. Beschreibung der Methode der Datenanalyse

SPSS: Die Veränderungen von Zeitpunkt 1 auf Zeitpunkt 2 und Zeitpunkt 3 werden mit Hilfe einer ITT mit hierarchischen mixed linear models berechnet.

2.12. Geplanter Beginn des Projekts

Die theoretische und konzeptuelle Vorbereitung der Studie läuft bereits. Mit der konkreten Vorbereitung der Therapien und dem Sampling der Stichprobe kann jederzeit nach Erteilung eines positiven Ethikbescheides begonnen werden; am liebsten ab Juni 2021

2.13. Voraussichtliche Gesamtdauer des Projekts

Gesamtdauer: 3 Jahre: Oktober 2020 - Oktober 2023
- Vorbereitungsphase: Oktober 2020 bis Mai 2021
- Studienzeitraum (Durchführung der Intervention, Prozessevaluation und Follow-up-Messung nach drei Monaten): August 2021 bis Dezember 2022
- Auswertung der Daten Dezember 2022 - Februar 2023
- Erstellung der Publikationen nach Abschluss der Datenerhebung ab Februar 2023
- Geplante Einreichung der angestrebten kumulierten Dissertation: September 2023

2.14. Finanzierung des Projekts?

keine

3. ProjektteilnehmerInnen

3a. Rekrutierung und Ausschluss

3.1. Geplante Anzahl der TeilnehmerInnen

Laut Power Berechnung mindestens N=165 Teilnehmner*innen (s.o.)

3.2. Voraussichtliche Dauer der Teilnahme an dem geplanten Projekt für die einzelnen TeilnehmerInnen (Dauer der Studientermine; Zeitraum)

24-26 Wochen gesamt: Intervention: 12 Wochen mit je einer 90 bis 100-minütigen Einheit und ca. 45 Minuten Hausübungen pro Woche; Follow-up weitere 12 Wochen.

3.3. Charakterisierung der TeilnehmerInnen

- Mindestalter: 18 Höchstalter: 65
- nicht persönlich Einwilligungsfähige einschließbar?

  Ja ☐ Nein ■

- Einschließbar sind

  ■ männliche und/oder ■ weibliche TeilnehmerInnen

3.4. Beschreibung des Rekrutierungsverfahrens (alle zur Verwendung bestimmten Materialien, z. B. Inserate, beilegen):

Zeitungsinserate (wurden noch nicht erstellt), Inserate in Online-Foren, Uni-interne Aussendungen, Kontakte zu Psychiater*innen und Psychotherapeut*innen, Kontakte zu Selbsthilfegruppen, Facebook-Gruppen, Psychologie heute

3.5. Legen Sie kurz die Auswahl der TeilnehmerInnen und Ein- und Ausschlusskriterien dar (Stichprobenverfahren, Begründung, Fallzahlschätzung) (explizite Begründung für den Einschluss von Personen aus geschützten Gruppen, z.B. Minderjährigen, temporär oder permanent nicht entscheidungsfähigen Personen; wenn zutreffend)

Stichprobengröße, wie oben dargelegt, errechnet mittels Power Analyse (G*Power) Kriterien; p <.05; 1-Beta = .80; ES (between): d=0.15 - 0.30; 3 Gruppen, 3 MZP. Benötigtes N=165 Personen, 55 pro Gruppe

Stichprobengewinnung: ad hoc/Gelegenheitsstichprobe; eine andere Ziehung ist in diesem Kontext nicht möglich.

Einschluss geschützter Gruppen nicht vorgesehen.

Einschlusskriterien:

-Alter: 18 bis 65

-Diagnose einer der folgenden Angststörungen: F40.1: Soziale Angststörung/ soziale Phobie; F41.0 Panikstörung mit oder ohne Agoraphobie, F41.1 Generalisierte Angststörung

-ausreichende Deutschkenntnisse

-durchschnittliche intellektuelle Leistungsfähigkeit (Einschätzung während des telefonischen/videogestützten Screenings)

-ausreichende Zeit für die wöchentlichen Einheiten und Hausaufgaben

-Akzeptanz des Studienprotokolls

Ausschlusskriterien:

-eine gleichzeitig oder in den folgenden drei Monaten geplante Teilnahme an einer psychotherapeutischen und/oder psychologischen Behandlung oder Beratung sowie eine Teilnahme an sonstigen psychologischen Gruppenangeboten

-Symptome einer schweren Depression, einer bipolaren affektiven Störung oder Manie (aktuell sowie anamnestisch), psychotische, schizophrene und/oder schizoaffektive Störungen, chronische Trauerreaktion, schwere Anorexie oder Bulimie, Substanzabhängigkeiten (Alkohol, illegale Drogen)

- schwere Persönlichkeitsstörungen, insbesondere Borderline-, narzißtische, anti-/ dissoziale und paranoide Persönlichkeitsstörung
- akute Suizidalität
- Umstellung, Dosisveränderung oder das vollständige Absetzen von Psychopharmaka in den vergangenen oder folgenden drei Monaten
- Fehlende Entscheidungsfähigkeit

3.6. Wird die Zustimmung der TeilnehmerInnen eingeholt (oder deren gesetzliche VertreterIn, wenn zutreffend)?

- [x] Ja (Einwilligungserklärung beilegen)
- [ ]

Nein

Wenn nein, warum nicht:

3.7. Nähe- bzw. Abhängigkeitsverhältnis zwischen TeilnehmerInnen und VersuchsleiterInnen (z. B. StudentIn–DozentIn, DienstnehmerIn–DienstgeberIn, etc.). Ist in diesem Zusammenhang Freiwilligkeit gewährleistet?

Es besteht kein Abhängigkeitsverhältnis

3.8. Wie vulnerabel sind die TeilnehmerInnen aus Sicht des/der Einreichers/in?

Bei den Proband*innen handelt es sich um Menschen mit erhöhter psychischer Belastetheit (Soziale Angststörung, Generalisierte Angststörung, Panikstörung), dementsprechend sind sie vulnerabler als psychisch unauffällige Menschen, jedoch weniger vulnerabel als Personen mit schweren psychotischen, affektiven und/oder Persönlichkeitsstörungen, welche aus der Studie ausgeschlossen werden. Insofern ist ihr klinischer Vulnerabilitätsgrad als mittelgradig einzustufen.

3b. Datenschutz

3.9. Welche personenbezogenen Daten werden erhoben?

Name, Wohnort, E-Mailadresse, Telefonnummer, Alter, Geschlecht, Familienstand, Bildung, Nationalität, psychometrische Daten aus den klinischen Verfahren, vergangene Psychotherapie/n, psychiatrische Behandlung, Einnahme von Psychopharmaka

3.10. Wie soll die Anonymität der TeilnehmerInnen gewährleistet werden?

Name, E-Mailadresse und Telefonnummer werden für organisatorische Zwecke und für die Übersendung der Links für die Online-Datenerhebung benötigt. Diese Daten werden unabhängig von den anderen - studienbezogenen - Daten nur von der Studienleiterin in einer Excel-Tabelle erfasst und in einem passwortgeschützten Laptop aufbewahrt. Andere Personen haben keinen Zugang dazu. Alle anderen Daten werden pseudonymisiert über einen Versuchspersonencode, der von den Patient*innen selbst zu erstellen ist, erfasst (= individueller Code).

3.11. Wenn eine vollständige Anonymisierung nicht möglich ist, wie wird die Privatsphäre geschützt?

Die Anonymität ist auf verschiedenen Ebenen zu schützen. 1. auf der Ebene der Behandler*innen wird sie durch die Berufsgesetze geschützt. Die Behandler*innen unterliegen der Verschwiegenheitsverpflicht nach dem österreichischen Psychologengesetz und dem deutschen Psychotherapeutengesetz. Entsprechend dürfen keinerlei Informationen über die Personen und deren Behandlung nach außen getragen werden. Lediglich im Zuge der Supervision und Intervision dürfen die Inhalte der Einheiten anonym besprochen werden. Aber auch die Supervisor*innen unterliegen den gleichen Berufsgesetzen wie die Behandler*innen, so dass auch auf dieser Ebene die Daten der Proband*innen geschützt sind.

Auf der Ebene der Forschungsdaten ist aufgrund der Pseudonymisierung mittels individueller Codes eine Zuordnung der Daten zu einer bestimmten Person nicht möglich; es sei denn die Person gibt ihren individuellen Code bekannt.

Auf der Ebene der konkreten Personendaten ist die Anonymität dadurch gewährleistet, dass über diese Daten nur die Studienleitung verfügt und nur dieser sind diese bekannt; allerdings kann auch sie keine Zuordnung der Namen zu den Daten herstellen, da sie nicht über die individuellen Codes verfügt. Die Personendaten sind durch eine passwortgeschützte Tabelle in einem ebenfalls geschützten Laptop nur ihr zugänglich.

3.12. Wie ist den TeilnehmerInnen die Einsicht in ihre persönlichen Daten möglich?

Die Teilnehmer*innen können nur über eine Offenlegung des individuell erstellten Codes Einsicht in ihre persönlichen Daten bei der Studienleitung beantragen. Individuelle Auskünfte und Einsichten in die Daten und die individuellen Ergebnisse der Studie können erst nach Abschluss der Studie in individuellen Rückmeldeterminen gegeben werden. Dafür müssen die Proband*innen jedoch einen Antrag an die Studienleiterin stellen.

3.13. Können sich TeilnehmerInnen über die Forschungsergebnisse informieren?

■ Ja ☐ Nein

Wenn nein, Begründung:

3.14. Wie und wie lange können TeilnehmerInnen die Löschung ihrer Daten verlangen?

Auf Verlangen und mit Bekanntgabe Codes des/r individuellen Teilnehmers*in können die Daten gelöscht werden. Die Löschung der Daten kann bis zum Abschluss der Datenerhebung beantragt werden. Anschließend werden sie in einem passwortgeschützten Rechner für weiterführende Analysen und Reanalysen gespeichert.

3.15. Die Datenverarbeitung erfolgt:

☐ personenbezogen, Begründung:

■ indirekt personenbezogen

Wie erfolgt die Anonymisierung?

Es erfolg eine Pseudonymisierung über individuell generierte Codes nach einem sechsstelligen Schlüssel

3.16. Werden Stimmen, Ton, Bilder oder Videos aufgenommen?

☐ Ja ■ Nein

wenn ja: Einwilligung der TeilnehmerInnen zur Aufnahme?

Ja

3.17. Was tun Sie, um zu gewährleisten, dass die TeilnehmerInnen aus der Studie aussteigen können oder die eigenen Daten zurückziehen können?

Schon im Vorfeld und im Zusammenhang mit der Rekrutierung werden die Teilnehmer*innen darüber aufgeklärt, dass sie mittels eines Schreibens (E-Mail) an die Studienleitung ihre Teilnahme an der Studie jederzeit zurückziehen bzw. beenden können. Damit werden sie mit sofortiger Wirkung aus derselben ausgeschlossen. Den Teilnehmer*innen wird zu Beginn eine

Einverständniserklärung vorgelegt bzw. per E-Mail zugesandt (siehe Anhang), in der dieses Vorgehen sowie alle weiteren Informationen zur Studie beschrieben sind. Sofern der Ausstieg mit einem Wunsch nach Löschung der individuellen Daten verbunden ist, muss der individuelle Code bei der Mitteilung (E-Mail) der Studienleitung bekannt gegeben werden.

3.18. Wie werden die Daten nach Beendigung des Projekts aufbewahrt und/oder vernichtet?

Die Daten werden elektronisch gespeichert und in einem verschlüsselten Ordner eines passwortgeschützten Laptops in pseudonymisierter Form aufbewahrt. Die Daten werden 30 Jahre gespeichert. Die Daten werden vollständig anonymisiert für weitere Forschung verwendet. In der Einverständniserklärung werden die Teilnehmer*innen über die Datenverarbeitung informiert und willigen dieser mit ihrer Unterschrift ein.

3c. Folgen für TeilnehmerInnen

3.19. Risiko- und Folgenabschätzung (z. B. Schmerzen, Unannehmlichkeiten, Verletzungen der persönlichen Integrität und Maßnahmen zur Vermeidung und/ oder Versorgung bei unvorhergesehenen/unerwünschten Ereignissen)

Grundsätzlich können unerwünschte Nebenwirkungen während einer Psychotherapie auftreten (Linden, & Strauß, 2018). Das Auftreten von unerwünschten Nebenwirkungen ist auch bei den hier geplanten Behandlungen nicht auszuschließen. Allerdings liegt das primäre Ziel der Behandlungen in der Reduktion der Angst- und allgemeinen psychopathologischen Symptomatik und in der Verbesserung des allgemeinen Wohlbefindens und der Lebensqualität. Nach Kenntnis der bisherigen Studienlage ist das Risiko für Unannehmlichkeiten und negative Entwicklungen im Bereich der PPT und der KVT als gering einzuschätzen. Die Kognitive Verhaltenstherapie ist bei der Behandlung von Angststörungen das Mittel der Wahl und konnte bisher sehr gute Effekte erzielen. Die Positive Psychotherapie zielt darauf ab, die Ressourcen und Stärken der Teilnehmer*innen zu stärken. Im Vorfeld der Behandlung werden die Teilnehmer*innen darüber aufgeklärt, dass sie, falls dennoch unerwünschte Effekte auftreten sollten, dies jederzeit ihren Behandler*innen mitteilen sollten. Sofern sich die negativen Veränderungen nicht reduzieren sollten, sollte die Studienleitung seitens der Patient*innen oder Behandler*innen kontaktiert werden. Zudem erhalten die Teilnehmer*innen die Rufnummer, für den für ihr Gebiet zuständigen Krisendienst ausgehändigt. Sollten im Rahmen der Behandlungen wiederholt Klagen über unerwünschte Erfahrungen oder Nebeneffekte der Therapien eingebracht worden sein, wird am Ende der Behandlung (T2) ein entsprechendes Verfahren zur systematischen Erfassung derartiger Ereignisse eingesetzt (z.B. INEP, Ladwig et al., 2014).

3.20. Welche Maßnahmen zur Prävention von Risiken werden getroffen?

Bei den Behandler*innen handelt es sich um Psychologen*innen (abgeschlossenes Masterstudium), die eine umfangreiche theoretische und praktische Erfahrung im Bereich Klinische Psychologie und Psychotherapie aufweisen. Zudem werden die Behandler*innen in die jeweiligen Manuale eingearbeitet und in diesen trainiert. Alle Therapieeinheiten werden aufgezeichnet, um die Manualtreue zu gewährleisten. Alle Behandler*innen erhalten regelmäßig Supervision und führen Intervision durch. Im Vorfeld an die Studie werden die nächstliegenden Krisendienste erfasst und die Teilnehmer*innen bekommen deren Telefonnummern und Erreichbarkeiten ausgehändigt. Zudem werden sie darüber informiert, dass sie über den gesamten Zeitraum der Studie hinweg in den Behandler*innen und der Studien- und Projektleitung professionele Ansprechpartner*innen zur Verfügung haben. Alle Teilnehmer*innen erhalten deren Kontaktdaten ausgehändigt. Vor Beginn der Intervention wird zudem erfasst, ob seitens der Teilnehmer*innen Suizidalität besteht oder eine schwere Depression vorliegt. Beides stellen Ausschlusskriterien für die Teilnahme an der Studie dar. Die Behandler*innen werden umfangreich darüber aufgeklärt, wie in einer Notfallsituation (Krise, Suizidalität) vorgegangen werden soll. Auch erhalten Sie in ihren Behandlungsmanualen entsprechende Checklisten abgedruckt, die ihnen helfen sollen im Falle einer Krise adäquat zu reagieren. Im Falle akuter Suizidalität wird zunächst mit den Teilnehmer*innen ein individueller Notfallplan erarbeite und es wird die Verschwiegenheitspflicht gemäß § 37 Psychologengesetz aufgehoben (dies wird von den Teilnehmern*innen im Zuge der Einverständniserklärung unterschieben) und die Behandler*innen wenden sich an die Studien- und/oder Projektleitung, mit der zusammen Lösungsmöglichkeiten erarbeitet werden.

3.21. Voraussichtliche Vorteile oder möglicher Nutzen für die eingeschlossenen TeilnehmerInnen

Aufgrund der bestehenden Forschungslage ist durch die Behandlung (sowohl PPT wie auch KVT) mit einer deutlichen Reduktion der Angst- und der allgemeinen psychischen Symptomatik und negativen Befindlichkeit zu rechnen (Laireiter & Furchlehner, 2018; Fava et al., 2005). Gleichzeitig wird vor allem bei den positiv-psychologischen Behandlungsbedingungen von einer starken Zunahme der positiven Befindlichkeiten, des Wohlbefindens und der Lebenszufriedenheit ausgegangen. Ein weiterer Nutzen der Teilnahme an der Studie ist, dass die Teilnehmer*innen die Behandlungen kostenlos erhalten werden, was angesichts der z.T. sehr lange Wartezeiten nach professioneller Hilfe ebenfalls als großer Benefit zu werten ist.

3.22. Methoden, um unerwünschte Effekte ausfindig zu machen, sie aufzuzeichnen und zu berichten (Beschreiben Sie wann, von wem und wie, z. B. freies Befragen und/oder an Hand von Listen)

In den wöchentlichen Therapiesitzungen können durch die Gespräche unerwünschte Effekte oder Nebenwirkungen der Behandlungen durch die Behandler*innen sofort erkannt werden. Die Behandler*innen sind im Rahmen ihrer Protokollführung und Dokumentation angehalten nicht nur den Verlauf der Sitzung und der Interventionen zu dokumentieren, sondern auch wahrgenommene Effekte und Rückmeldungen der Teilnehmer*innen (insbesondere negative und problembezogene). Im Fall der Kommunikation einer negativen Entwicklung ist dies besonders zu dokumentieren; sollte die negative Entwicklung als problematisch oder gefährlich eingestuft werden, ist mit dem/der betreffenden Teilnehmer*in zeitnah ein individuelles Gespräch geführt werden. Kann darin keine adäquate Lösung gefunden werden, ist die Studien- und/oder Projektleitung zu kontaktieren, um eine Lösung zu erarbeiten (s. auch oben). Zusätzlich bieten die Intervision und die Supervision der Behandler*innen Raum, um mögliche Probleme und problematische Entwicklungen bei einzelnen Teilnehmer*innen zu reflektieren und fachlichen Rat von ausgebildeten Klinischen Psycholog*innen/Psychotherapeut*innen einzuholen. Zudem ist es den Patient*innen im Falle von Belastungen oder unerwünschten Entwicklungen außerhalb der oder zwischen den Behandlungssitzungen jederzeit möglich sich per E-Mail mit die Behandler*innen in Verbindung zu setzten oder sich bei der Studien- oder Projektleitung zu melden.

3.23. Falls notwendig: Plan zur Behandlung und/oder Versorgung, nachdem die Personen ihre Teilnahme an dem Projekt beendet haben (wer wird verantwortlich sein und wo)

Sollten Probleme oder negative Effekte nach Beendigung der Behandlungen - im Follow-up-Zeitraum oder auch danach - auftreten, werden die Teilnehmer*innen gebeten sich mit der Studien- oder Projektleitung in Verbindung zu setzen (es können aber auch die Behandler*innen kontaktiert werden). Bei Bedarf können die Teilnehmer*innen in diesem Fall ein Beratungsgespräch mit diesen Personen in Anspruch nehmen. Dabei sollen die Probleme geklärt und individuelle Lösungen erarbeitet werden. Auch ist zu eruieren, ob die Probleme auf die zuvor absolvierte Behandlung zurückzuführen ist. In jedem Fall ist das Gespräch zu dokumentieren. Die angesprochenen Problemlösungen könnten u.a. darin bestehen, dass die/der Betroffene darin unterstützt wird Hilfe bei einem/r Psychotherapeut*in in der Region zu suchen, selbst ihren Hausarzt oder einen Facharzt aufsucht, oder - im schlimmsten Fall - eine Kriseninterventionsstelle oder medizinische Einrichtung in Anspruch nimmt, sofern eine Indikation dafür vorliegt. Es könnte ggf. aber auch noch eine weitere untersützende Intervention seitens der Studien- oder Projektleitung angeboten

werden. Beide sind ausgebildete bzw. sich in Ausbildung befindliche Psychotherapeut*innen.

3.24.Betrag und Verfahren zur Vergütung der TeilnehmerInnen (Höhe des Betrags sowie wofür dieser bezahlt wird; z. B. Fahrtspesen, Einkommensverlust usw.)

Es ist keine Vergütung der Teilnehmer*innen vorgesehen; die Teilnehmer*innen erhalten eine kostenlose psychologische Behandlung als indirekte und immaterielle Vergütung (s.o.)

3.25.Betrag und Verfahren zur Entschädigung der TeilnehmerInnen (Höhe des Betrags sowie wofür dieser bezahlt wird; z. B. Schmerzen usw.)

Die Teilnehmer*innen erhalten keine Entschädigung; es werden keine aversiven Stimuli appliziert oder Schwerzen verursacht.

3d. Weitere ethische Aspekte

3.26.Werden die TeilnehmerInnen in vollem Umfang über Art, Ziel und Inhalt des Projekts informiert?

■ Ja ☐ Nein, Begründung:

3.27. Werden die TeilnehmerInnen getäuscht?

☐ Ja ■ Nein

Wenn Ja, Beschreibung und Begründung:

3.28.Ethische Überlegungen: Identifizieren und beschreiben Sie alle möglicherweise auftretenden Probleme

Da sich die Behandler*innen (erst) in Ausbildung zur/m Klinischen Psycholog*in bzw. Psychologische*n Psychotherapeuten*in befinden und diese noch nicht abgeschlossen haben, fehlt es ihnen womöglich an einer vollumfänglichen Erfahrung und einem breiten Methodenrepertoire psychologischer Behandlung, um mit allen möglicherweise auftretenden Problemen umzugehen.
Dies kann durch folgende Komponenten kompensiert werden:
1. vollständig ausformulierte und ausführliche Manuale,
2. eine intensive Einschulung mit Selbsterfahrung,
3. einer begleitenden kontinuierlichen Intervision, bei Bedarf unter Beisein der Studienleiterin oder - falls gewünscht - des Projektleiters
4. einer begleitenden regelmäßig stattfindenden Supervision durch klinisch erfahrene Expert*innen in PPT und/oder KVT,
5. dem Verfügen über praktische Erfahrung aus eigenen Praktika und beruflichen Tätigkeiten in der Psychiatrie oder Psychosomatik und der Behandlung ambulanter Patienten*innen im Rahmen ihrer Ausbildungen und
6. dem Verfügen über ausreichendes Wissen auf dem Gebiet der Klinischen Psychologie, Psychotherapie und Positiven Psychologie.

Es könnte passieren, dass sich Behandler*innen im Verlauf der Intervention überfordert fühlen, dies sollte im Rahmen der Intervision und/oder Supervision rasch erkannt und dafür jeweils eine adäquate individuelle Lösung gefunden werden (z.B. Wechsel eines/r oder beider Behandler*innen). Bei einem Wechsel der Behandler*in wird die weitere Versorgung der Patient*in rechtzeitig sichergestellt. Im Falle eines Wechsels übernimmt eine andere Behandler*in, welche ebenfalls intensiv in den Manualen geschult wurde, die Behandlung. In diesem Fall erfolgt eine kontrollierte Übergabe.

Zudem werden alle Behandler*innen nach folgenden strengen Kriterien ausgewählt. Diese sind:

- abgeschlossenes Psychologie Masterstudium
- Aktuell in der Ausbildung zur/zum Psychologische*n Psychotherapeuten*in oder Klinischen Psychologen*in
- Klinische Erfahrung in Bereich Psychiatrie/Psychosomatik mit Erwachsenen
- Positive Psychologie Vorlesungen oder Seminare im Studium belegt
- ausreichend Zeit, um regelmäßige Einheiten abhalten zu können (Min 6Std. pro Woche)

Sollte es trotz umfassender Screenings- und Eingangsdiagnostik doch zu einer suizidalen Krise kommen, wird der/die Patient*in ggf. weiterverwiesen und/oder eine Krisenintervention eingeleitet.

Auch könnte der Eindruck entstehen, dass mit dieser Studie eine Psychotherapie angeboten wird. Um diesen Eindruck zu verhindern, werden die Teilnehmer*innen von Anfang an darüber informiert, dass es sich bei diesem Projekt nicht um eine (Kassen-)Psychotherapie oder eine psychologische Behandlung, die von niedergelassenen Psychotherapeut*innen durchgeführt wird und die Behandler*innen keine abgeschlossene Ausbildung zum/r klinischen Psychologen*in und/oder Psychotherapeuten*in besitzen. Die Teilnehmer*innen unterschreiben die Aufklärung darüber und deren Kenntnisnahme in der Einverständniserklärung.

3.29. In welchem Verhältnis stehen potentielle Risiken der Studie zum erwarteten Nutzen?

Nach unserer Einschätzung überwiegt der erwartete Nutzen der Studienteilnahme eindeutig die Risiken. Sowohl die Teilnehmer*innen (Reduktion der Angstsymptomatik und Verbesserung des allgemeinen Wohlbefindens), die Forschung (wichtige Erkenntnisse in einem jungen Forschungsfeld), als auch

die Gesellschaft (neue Wirkmechanismen zur Steigerung des Wohlbefindens werden evaluiert) profitieren von diesem Projekt.

3.30. Wann ist lt. Risikoplan ein Aussetzen des Projekts vorgesehen? Unter welchen Umständen wird das Projekt abgebrochen?

Wenn sich bei mind. 10% der Teilnehmer*innen negative Effekte zeigen, wird die Behandlung unterbrochen. Sollten mehr als 15 % eine Verschlechterung berichten, wird die Studie umgehend unterbrochen und die Ursache dafür eruiert. Sollte es zu einem Abbruch der Studie kommen, werden die Teilnehmer*innen von erfahrenen Psychotherapeuten*innen in einer Einzelsitzung betreut und sie werden bei Bedarf bei der Suche einer weiteren Behandlung/ Psychotherapie unterstützt.

3.31. Ist eine Versicherung erforderlich?

☐ Ja ■ Nein

Wenn ja: Versicherungsgesellschaft

4. Sonstige Anmerkungen

Die erstellten Manuale entsprechen dem Standard psychologischer Behandlungsmanuale und wurden aus bestehenden und evaluierten Manualen abgeleitet bzw. stellen Ausarbeitungen bzw. Verkürzungen bestehender Manuale dar. Außerdem wurden sie von fachkompetenten Expert*innen der PPT und der KVT begutachtet und für geeignet befunden

5. Projektteam

Geben Sie alle MitarbeiterInnen des Projekts an:

Name	Institution	Funktion*	Qualifikation**
a.o. Univ. Prof. Dr. Anton Rupert Laireiter	Fachbereich Psychologie, Universität Salzburg	ProjektleiterIn***	a.o. Univ.-Prof. Klinischer Psychologe, Psychotherpeut; Fortbildungen in Positiver Psychologie

Catiana Luisa Engelhardt	Fachbereich Psychologie, Universität Salzburg	Doktorandin, Erstellung der Manuale, Planung und Auswertung der Studie	Psychologin (MSc), Doktoratsstudium Universität Salzburg, Psychologische Psychotherapeutin i.A., zertifizierte Anwenderin und Beraterin für Positive Psychologie durch die Deutsche Gesellschaft für Positive Psychologie (DGPP) Berlin
Mag. Brigitte Schweiger-Schrader	Fachbereich Psychologie, Universität Salzburg	Supervisorin	Klinische Psychologin, Lehrbeauftragte und Trainerin in Positiver Psychologie
N.N.		Supervisiorin	Trainerin in Positiver Psychologie

*z. B. Planung, Auswertung, Leitung, Datenerhebung
** z. B. Prof., Senior Scientist, DissertantIn, Postdoc
*** ProjektleiterIn sollte in dem Forschungsgebiet promoviert sein

Gibt es Interessenskonflikte von beteiligten ForschungsmitarbeiterInnen?

☐ Ja ■ Nein

Wenn ja: Beiblatt Interessenskonflikt ausfüllen und beilegen

6. Name und Unterschrift des Antragstellers/der Antragstellerin

Name: Catiana Luisa Engelhardt
Institution/Firma: Fachbereich Psychologie, Universität Salzburg

Position: Doktorandin

Unterschrift des Antragstellers/der Antragstellerin: Hiermit bestätige ich, dass die in diesem Antrag gemachten Angaben korrekt sind und ich der Meinung bin, dass die Durchführung des Projekts in Übereinstimmung mit nationalen Regelungen und mit den Prinzipien der guten wissenschaftlichen Praxis möglich sein wird.

____

Unterschrift des Antragstellers/der Antragstellerin, Datum

Falls es sich bei dem Projekt um eine Dissertation (PhD-Arbeit) oder Masterarbeit bzw. Diplomarbeit handelt: **Name und Unterschrift des Betreuers/der Betreuerin**

Name: a.o. Univ.-Prof. i.R. Dr. Anton-Rupert Laireiter
Institution/Firma: Fachbereich Psychologie, Universität Salzburg
Position: a.o.Univ.-Prof. i.R., Priv.Doz. Schwerpunkte u.a.: Psychotherapie, Positive Psychologie

Unterschrift des Betreuers/der Betreuerin: Hiermit bestätige ich, dass die in diesem Antrag gemachten Angaben korrekt sind und ich der Meinung bin, dass die Durchführung des Projekts in Übereinstimmung mit nationalen Regelungen und mit den Prinzipien der guten wissenschaftlichen Praxis möglich sein wird.

____

Unterschrift des Betreuers/der Betreuerin, Datum

Beizulegen sind:

- Projektantrag
- Studienteilnehmerinformation/Einverständniserklärung
- Kurzer akademischer Lebenslauf/Qualifikationsnachweis der antragstellenden Person(en)
- Beiblatt Interessenskonflikt (falls zutreffend)
- Bereits vorhandene Voten von Ethikkommissionen (falls zutreffend)
- TeilnehmerInnenversicherung (falls nötig)
- In der Studie zur Datenerhebung verwendete Fragebögen, Interviews, etc.
- Anzeigen, Broschüren etc. zur Teilnehmerrekrutierung
- Check-Liste
