# Supplementary material for: Extent of therapeutic support in Positive Psychotherapy: A randomized comparative efficacy study for the treatment of anxiety disorders in an online group setting
Source: PLoS One. 2026 Jul 28;21(7):e0354083. doi: 10.1371/journal.pone.0354083 (PMC13411940; doi:10.1371/journal.pone.0354083)
Supplement: S2 Protocol — English translation of the study protocol as approved by the ethics committee. (PDF) [file pone.0354083.s002.pdf]

Application form

to Evaluation of a project by the Ethics Committee of the University of Salzburg

Version 3. (October zo19)

Space for receipt stamp, EK number, etc. Please leave blank!

Please answer the individual questions with the essential information (usually max. 15s words, except for 2 3) Please answer non-applicable points with t.n.z. (does not apply). The application form must be completed in German!

1. general

1.1. Project title

Online Intervention Study in Anxiety Disorders with Cognitive Behavioral Therapy and Positive Psychology.

1.2. Applicant

Catiana Luisa Engelhardt, M.Sc.

1.3. The study presented is: a project financed by

third parties

University internal project number/cost center (if applicable):

t.n.z.

an internal university project a

dissertation (PhD thesis) a

master's thesis/diploma thesis

Name of the supervisor:

a.o. Univ.-Prof. Dr. Anton- Rupert Laireiter

Submission was requested By:

Supervisor Study law body

1.4. Why is a decision of the ethics committee required (specify publication organ in case of publication)?

This is a study of an intervention with patients suffering from an anxiety disorder (generalized anxiety disorder, social anxiety disorder/social phobia and/or panic disorder with or without agoraphobia). The publication of three papers is planned (publication organ not yet known)

1.5. Has the project already gone through a review process?

Yes NoIf yes, at which institution? ten enclose!

Gutach-

1.6. Are there already votes from other ethics committees?

Yes No If yes, attach votes!

2. structured summary of the project

2.1. Project type

This is an online intervention study in a group setting with an efficacy and a process study. Cognitive Behavioral Therapy and Positive Psychotherapy are compared.

2.2. Subject

Clinical psychology, psychotherapy, positive psychology

2.3. General summary of the project (background, questions, methods, max. 500 words)

In the planned study, a randomized controlled comparative study will be conducted in anxiety patients with generalized anxiety disorder, social

Anxiety disorder/social phobia and/or panic disorder with or without agoraphobia with two active treatment conditions (positive psychotherapy and cognitive behavioral therapy) and a comparison group (online positive psychotherapy with minimal therapeutic supervision) in a group setting in an online format (tea groups). Included in this study is also a process efficacy study to determine effect moderating (effective) factors.1 Positive Psychotherapy, which originated from Positive Psychology, has set itself the goal of introducing a positive focus into psychotherapeutic work. A new treatment manual, based on that of Rashid and Seligman (2018), was developed for Positive Psychotherapy. For Cognitive Behavioral Therapy, a new - superordinate (applicable to all three anxiety disorders) - treatment manual was also developed based on that of Rashid and Seligman (2018).

"Therapy Tool for Anxiety Disorders" by Hagena and Gebauer (2014). The treatment manual of the control group is thematically based on that of Positive Psychotherapy, but strongly shortened. The treatment manuals of PPT and KVT each comprise 12 group sessions of 90-100 minutes each and one individual session. The treatment manual of the control group comprises 12 sessions, three of which (the first, the sixth and the twelfth) take place in an online group setting, and the remaining nine are self-completed with the help of instructional videos and e-mail communication. All therapy sessions take place online. The individual groups consist of six to eight participants. The participants in each group also have access to a website created specifically for the study. This website provides work materials, audio files and instructional videos. In addition, it offers the possibility of contacting the study director via a chat function; it also serves as a virtual bulletin board to make certain exercises visible in anonymized form. The study includes three measurement time points, a pre- and post-measurement and a follow-up after three months. The treatments are carried out by psycho1ogists in training as clinical psycho1ogists and psychological psychotherapists in Austria and Germany. All psycho1ogists are intensively trained in the respective manuals, participate in regular intervision sessions and receive supervision from experienced representatives of Positive Psychology and Cognitive Behavioral Therapy at regular intervals. All group sessions are recorded and discussed in the supervision. Each session is checked for adherence to the manual. As mentioned above, the study also includes a process efficacy study. In this study the intra-session processes as well as the inter-session processes of the therapy with Positive Psychotherapy and Cognitive Behavioral Therapy will be evaluated and related to the therapy outcome. Intra-session processes are understood as all those changes that occur directly in response to specific events in the therapy session, whereas inter-session processes are defined as processes of change between two sessions that are brought about by memories and representations of the session (Or- linsky & Geller, 1993). Intra-session processes will be assessed with the help of the Berner Stundenbeurteilungsbogen íiir Patienten (Flückiger et al., 2010), where patients will be asked after each session whether they have experienced it and whether they think it has brought progress, on the one hand in the sense of the

The intersession processes are recorded with the help of the Hartmann and Orlinsky Intersession Questionnaire. The intersession processes are recorded with the help of the Hart- mann and Orlinsky intersession questionnaire. In addition to the recording of the processes, it is to be examined in particular to what extent Positive Psychotherapy and Cognitive Behavioral Therapy differ in the therapy processes (intra- and intersession processes) and which variables of both process types predict the therapy success. The control group is not included in the process study. It serves as a comparison group for the effectiveness study in order to examine the effect of a low-threshold Positive Psychology intervention compared to a guided intervention. In order to examine the effect of process variables on therapy outcome (decrease in anxiety symptomatology and general psy- chic distress, increase in happiness experience, life satisfaction and quality of life), two global indicators are formed: 1. frequency and intensity of intra- and intersession activities and 2. emotional valence of intra- and intersession processes (positive vs. negative). The aim of this study is to identify variables of change, to gain knowledge about the therapy processes and thus to optimize the therapy process.

2.4. Objective, questions, hypotheses (if applicable).

The effectiveness study aims to clarify the extent to which positive psychotherapy compared to cognitive behavioral therapy and a control group (online positive psychotherapy with minimal therapeutic support - hereafter referred to as "minimally supported online PPT") in patients with generalized anxiety disorder, social anxiety disorder/social phobia and/or panic disorder with or without agoraphobia (hereinafter referred to for brevity as "anxiety patients") can reduce anxiety symptoms and increase overall life satisfaction, quality of life, and happiness. Through this study, the effectiveness of Positive Psychotherapy in the treatment of anxiety disorders will be evaluated for the first time in an online format.

Research hypotheses:

1) The anxiety patients* who participated in the Positive Psychotherapy treatment program showed a significant decrease in anxiety symptomatology between pre- and postmeasurement.
2) Anxiety patients who participated in the Positive Psychotherapy treatment program showed a significantly greater decrease in anxiety symptoms compared to patients who participated in the minimally supervised online PPT treatment program.
3) The anxiety patients* who participated in the Cognitive Behavioral Therapy treatment program showed a significant decrease in anxiety symptomatology between pre- and postmeasurement.

4) Anxiety patients who participated in the Cognitive Behavioral Therapy treatment program showed a significantly greater reduction in anxiety symptoms compared to patients who participated in the minimally supervised online PPT treatment program.
5) The anxiety patients who participated in the Positive Psychotherapy treatment program showed a comparable decrease in anxiety symptoms as the patients who participated in the Cognitive Behavioral Therapy treatment program.
6) Anxiety patients who participated in the Positive Psychotherapy treatment program experience a significant increase in happiness between pre- and postmeasurement through direct influence on positive emotions, on recognizing and using one's strengths, and on experiencing meaning.
7) The anxiety patients* who participated in the Positive Psychotherapy treatment program experience a significantly greater increase in happiness experience through direct influence on positive emotions, on recognizing and using one's strengths, and on experiencing meaning compared to the patients* who participated in the minimally supervised on line PPT.
8) The anxiety patients who participated in the Positive Psychotherapy treatment program experience a significantly greater increase in happiness experience compared to the patients who participated in the Cognitive Behavioral Therapy treatment program through direct influence on positive emotions, on recognizing and using one's strengths, and on experiencing meaning.
9) Anxiety patients who participated in the Positive Psychotherapy treatment program experience a significant increase in life satisfaction and quality of life between pre- and postmeasurement by directly influencing positive emotions, recognizing and using their strengths, and experiencing meaning.
10) Anxiety patients who participated in the Positive Psychotherapy treatment program experience a significantly greater increase in life satisfaction and quality of life than patients who participated in the minimally supervised online PPT treatment program by directly influencing positive emotions, recognizing and using one's strengths, and experiencing meaning.
11) Anxiety patients who participated in the Positive Psychotherapy treatment program experience a significantly greater increase in life satisfaction and quality of life than patients who participated in the Cognitive Behavioral Therapy treatment program by directly influencing positive emotions and self-compassion, recognizing and using one's strengths, and experiencing meaning.

12)The significant decrease in anxiety symptoms in the patients who participated in the Positive Psychotherapy treatment program remains stable over a period of three months.
13)The significant decrease in anxiety symptoms in patients who participated in the Cognitive Behavioral Therapy treatment program remains stable over a period of three months.
14)The significant increase in happiness among patients who participated in the Positive Psychotherapy treatment program remained stable over a period of three months.
15)The significant increase in life satisfaction and quality of life among patients who participated in the Positive Psychotherapy treatment program remained stable over a period of three months.
16)In terms of an exploratory question, it is assumed that the effects of Online Positive Psychotherapy with minimal therapeutic supervision will not remain stable over three months.

The process study will evaluate the process of therapy in Positive Psychotherapy and Cognitive Behavioral Therapy. In the process study, both the intra-session processes and the inter-session processes of therapy with Positive Psychotherapy and Cognitive Behavioral Therapy are to be evaluated. Furthermore, it will be determined which process variables (intra-session or inter-session processes) predict the success of therapy and to what extent Positive Psychotherapy and Cognitive Behavioral Therapy differ in the therapy processes. The intra-session processes will be assessed with the help of the hourly patient assessment form (STU-P). The participants will fill out this questionnaire in digital form after each session. Intersession processes are to be recorded with the help of the Intersession Questionnaire (ISF). The participants fill out this questionnaire in digital form before each session. The aim is to examine and evaluate the process. In this way, it may be possible to make changes to the manuals even after the end of the study and to gain insights into the therapeutic processes. The aim is to identify therapeutic process variables and thus optimize the therapy process.

Research hypotheses:
1) Anxiety patients whose intersession activity is emotionally positive show a greater decrease in their anxiety symptoms than patients whose intersession activity is negative. There is a significant correlation between the emotional occupation of the intersession processes and the decrease in anxiety symptoms.
2) Anxiety patients whose intersession activity is emotionally positive show a stronger increase in their happiness experience than patients whose intersession activity is negative. There is a significant correlation between the emotional occupation of the intersession processes and the increase in happiness.

3) Anxiety patients whose intersession activity is emotionally positive show a greater increase in their life satisfaction and quality of life than patients whose intersession activity is negative. There is a significant correlation between the emotional involvement of the intersession processes and the increase in life satisfaction and quality of life.
4) Anxiety patients whose intra-session activity has positive emotional connotations show a greater decrease in their anxiety symptoms than patients whose intra-session activity has negative connotations. There is a significant correlation between the emotional content of the intra-session processes and the decrease in anxiety symptoms.
5) Anxiety patients whose intra-session activity is emotionally positive show a stronger increase in happiness than patients whose intra-session activity is negative. There is a significant correlation between the emotional content of the intra-session processes and the increase in happiness.
6) Anxiety patients whose intra-session activity is emotionally positive show a higher increase in their life satisfaction and quality of life than patients whose intra-session activity is negative. There is a significant correlation between the emotional occupation of the intra-session processes and the increase in life satisfaction and quality of life.
7) Anxiety patients who show a higher level of intersession activity show a greater decrease in their anxiety symptoms at the end of therapy than those who show a lower level of intersession activity. There is a significant positive correlation between the intensity and frequency of intersession activities and the decrease in anxiety symptoms.
8) Anxiety patients who exhibit a higher level of intersession activity show a greater increase in their happiness at the end of therapy than those who exhibit less intersession activity. There is a significant positive correlation between the intensity and frequency of intersession processes and the increase in happiness.
9) Anxiety patients who exhibit a higher level of intersession activity show a greater increase in their life satisfaction and quality of life at the end of therapy than those who exhibit less intersession activity. There is a significant positive correlation between the intensity and frequency of intersession processes and the increase in life satisfaction and quality of life.
10) Anxiety patients who show a higher level of intra-session activity show a greater decrease in anxiety symptoms at the end of therapy than those who show less intra-session activity. There is a significant positive correlation between the intensity and frequency of intra-session processes and the decrease in anxiety symptoms.
11) Anxiety patients who exhibit higher levels of intra-session activity show a greater increase in happiness experience at the end of therapy.

bens, than those who have less intra- session activities. There is a significant positive correlation between the intensity and frequency of intra-session processes and the increase in happiness experience.

12) Anxiety patients who exhibit a higher level of intra-session activity show a greater increase in life satisfaction and quality of life at the end of therapy than those who exhibit less intra-session activity. There is a significant positive correlation between the intensity and frequency of intra-session processes and the increase in life satisfaction and quality of life.

2.5. Scientific and social relevance (rationale of the project) The scientific relevance of this study is very high. There are currently only a small number of studies that have investigated the effectiveness of Positive Psychotherapy in clinical patients. Most of the existing

The most recent studies were conducted on patients with depressive disorders. Here, a high efficacy of treatment with positive psychotherapy was found (e.g., Laireiter & Furchtlehner, 2018). The intended study would be the first to examine and comparatively evaluate the effectiveness of the procedures Cognitive Behavioral Therapy and Positive Psychotherapy for Anxiety Disorders in a group setting under online conditions. To the applicant's knowledge, the planned process study is the first ever to have been conducted on this topic in the context of Positive Psychotherapy. Its results can therefore provide important and directional findings with regard to therapeutic processes and changes in Positive Psychology. Also the social relevance is to be estimated as very high. Anxiety disorders are the most common mental disorders of all (Jacobi et al., 2004; Kessler et al., 2005).

According to international studies, lifetime prevalence ranges from 14 to 29% (Kessler et al., 2005; Somers et al., 2006). The quality of life of those affected is very severely limited by their disorder. According to the World Health Organization (WHO), anxiety disorders ranked sixth worldwide and fourth in highly developed (industrialized) countries among all mental and somatic disorders in terms of years lived with disability (YLD) in 2015. Compared to the previous, rather deficit-oriented psychotherapy, Positive Psychotherapy represents a very promising, innovative approach not only to reduce the symptoms, but above all to promote well-being, quality of life and life satisfaction.

2.6. Research Design

3x3 variance-analytic within-subjects design (within-subjects: repeated measures factor with three measurement time points: Pre, Post, Follow-up after three months). Between Subjects: Three treatment groups: two online.

conducted intervention and a low-level guided comparison group Con- trolled intervention study based on written treatment manuals; explicit inclusion and exclusion criteria (see below); rational sample design (see below); randomized patient assignment => RCT.

2.7. Description of the study design (survey time points, type and number of groups, control groups, etc.)

In the following, the procedures used are presented with the common abbreviations; a more precise description can be found below. The study comprises a total of five measurement time points, two scree- ning (pre-selection and telephone/video-assisted interview) and three the- rapy-related measurements.

1. Online pre-screening for general eligibility using GAD-7, BAI, FQ and PAS and demographic and clinical variables.

2. The second screening takes place in the form of a telephone or video call before the intervention begins. Here, the exclusion criteria are determined by means of

Mini-DIPS and PSS-K are reviewed. In addition, all patients will be informed comprehensively about the course of the study and its contents, and their explicit consent to participate in the study according to the study criteria will be obtained. All participants will also receive the telephone number of the crisis service responsible for their area, so that they can contact them in the event of an emergency.

4. The participants included in the study who meet the inclusion and exclusion criteria will be assigned to one of the three intervention groups (PPT, KVT, minimally supervised PPT). The allocation is ran- domized. Subsequently, they receive a link to the online questionnaire and complete the questionnaires online to collect the dependent baseline variables (T1). The following self-report questionnaires concerning anxiety are given: Beck Anxiety Inventory (BAI), Panic and Ago- raphobia Scale (PAS), Generalized Anxiety Disorder 7 (GAD-7), Social Anxiety and Social Skills Deficits Questionnaire (SASKO), Anxiety Questionnaire (AF)/Fear Questionnaire (FQ), Patient Health Questionnaire.

(PHQ-9), ICD-10- Symptom Rating (ISR) for the assessment of general psychic abnormality. Positive Outcomes: Positive Psychotherapy Inventory (PPTI), Flourishing Scale (FS), Satisfaction with Life Scale (SWLS); External Assessment: Based on the clinical interview, the following procedures are processed by the interviewer: MiniDIPS (mental disorders), PSS-K (personality disorders), SCID PD (personality disorders) Panic and Agoraphobia Scale (PAS) and Hamilton Anxiety Scale (HAMA).

5. The 12-week intervention based on the two manuals is conducted by psycho1ogists in training as clinical psycho1ogists or psychological psychotherapists in a dual trainer setting (two therapists). All therapists are intensively trained in

The group members are trained in the respective manuals, regularly update their work, and also receive regular supervision from experienced representatives of Positive Psychology and Cognitive Behavioral Theory. All group sessions are recorded and discussed in the supervision. Each session is evaluated for its adherence to the manual.

6. Before each therapy session, participants complete the Intersession Questionnaire (ISF) online for process analysis of the therapies, and after each session they complete the Patient Hourly Evaluation Form (STU-P) online.

7. The second questionnaire survey (T2) takes place after the last therapy session. For this purpose, the participants are sent the link to the online survey again. The same self-assessment procedures are used as for T1. This time, too, there will be an external assessment. This is done by the therapists using the Panic and Agoraphobia Scale (PAS) and the Hamilton Anxiety Scale (HAMA).

8. Three months after the end of the online groups, the participants receive another email with a link and a request to complete the questionnaire battery (=Fo1low-Up, T3). This time, too, the same self-assessment procedures are given as in TI and T2. At this point of measurement, no further external assessment takes place.

2.8. Description of the methods of data collection, instruments

As can be seen from the previous section, both self-assessment and external assessment procedures are integrated into the data collection. In both cases, psychometrically tested clinical interviews and scales are used. The collected characteristics of the efficacy study are divided into primary and secondary outcomes and include anxiety expression as well as additional psychopathological characteristics.

Personal and socio-demographic data:
- Name, place of residence, e-mail address, telephone number; age, gender, marital status, education, nationality.

Screening and diagnostics:
- MiniDIPS: (current and lifetime) diagnoses of mental disorders
- PSS-K: Personality Disorder Screening Short Form
- SCID PD: Structured Clinical Interview-Personality Disorders (for indications according to PSS-K).
- Other clinical data: psychometric data from clinical procedures, psychiatric treatment, use of psychotropic drugs, past psychotherapy/s (number, type).

Primary Outcomes:
1. Anxiety Symptomatology:

Self-assessment	BAI, PAS; GAD-7, FQ
External assessment	PAS, HAMA
2. positive outcome	
Positive Psychotherapy	PPTI
Inventory Flourishing Scale	FS
Satisfaction with Life-Scale	SWLS
Secondary Outcome:	
ICD-10 Symptom Rating	ISR
Patient Health	
	Questi
onnairePHQ-9	
Process Study:	
Hourly assessment questionnaire me patients	
STU-P Intersession questionnaire	ISF

2.9. Description of the sample (participants)

The sample size was calculated using a power analysis (G*Power). This amounts to 165 subjects; accordingly, 55 subjects would be included in each of the three intervention groups. Regardless, the following inclusion and exclusion criteria are set for the study:

Inclusion criteria:

- Age from 18 to 65
- Diagnosis of any of the following anxiety disorders: F40.1: Social anxiety disorder/social phobia; F41.0 Panic disorder with or without agoraphobia, F41.1 Generalized anxiety disorder.
- sufficient knowledge of German
- average intellectual ability (assessment during telephone/video-based screening)
- Sufficient time for the weekly sessions and homework assignments
- Acceptance of the study protocol

Exclusion Criteria:

- Participation in psychotherapeutic and/or psychological treatment or counseling planned at the same time or in the following three months, as well as participation in other psychological group offers
- Symptoms of major depression, bipolar affective disorder, or mania (current as well as history), psychotic, schizophrenic, and/

or schizoaffective disorder, acute grief reaction, severe anorexia or bulimia, substance dependencies (alcohol, illicit drugs)
- severe personality disorders, especially borderline, narcissistic, anti/dissocial and paranoid personality disorder
acute suicidality
- Switch, dose change, or complete discontinuation of psy- chotropic drugs in the past or following three months Lack of decision-making capacity.

2.10. Description of the survey locations

The screening as well as the intervention and the surveys take place in an online format. However, for the implementation of the online interventions, fixed locations are determined for the psycho1ogists. In Salzburg, the online interventions are conducted on the premises of the Counseling Center for Clinical Psychology, Psychotherapy & Health Psychology.

2.11. Description of the method of data analysis

SPSS: Changes from time point 1 to time point 2 and time point 3 are calculated using an ITT with hierarchical mixed linear models.

2.12. Planned start of the project

The theoretical and conceptual preparation of the study is already underway. The concrete preparation of the therapies and the sampling of the sample can be started at any time after a positive ethics decision has been issued; preferably from June 2021 onwards.

2.13. Estimated total duration of the project

Total duration: 3 years: October 2020 - October 2023
- Preparation phase: October 2020 to May 2021
- Study period (implementation of the intervention, process evaluation, and follow-up measurement after three months): August 2021 to December 2022
- Evaluation of data December 2022 - February 2023
- Preparation of publications after completion of data collection as of February 2023
- Planned submission of the targeted cumulative dissertation: September 2023.

2.14. Funding of the project?

none

3. project participants

3a. Recruitment and exclusion

3.1. Planned number of participants

According to Power calculation at least N=165 participants (see above)

3.2. Expected duration of participation in the planned project for individual participants (duration of the study dates; period)

24-26 weeks total: Intervention: 12 weeks, each with one 90- to 100-minute session and about 45 minutes of home exercises per week; follow-up another 12 weeks.

3.3. Characterization of participants

Minimum age: 18Maximum age : 65

not personally capable of giving consent includable?

Yes No

Includable are

male and/or female participants

3.4. Description of recruitment process (include all materials intended for use, e.g., advertisements):

Newspaper advertisements (have not yet been created), advertisements in online forums, internal university mailings, contacts to psychiatrists and psychotherapists, contacts to self-help groups, Facebook groups, Psychologie heute (Psychology Today).

3.5. Briefly describe the selection of participants and inclusion/exclusion criteria (sampling method, rationale, caseload estimate) (explicit rationale for inclusion of persons from protected groups, e.g., minors, temporarily or permanently incapacitated persons; if applicable).

Sample size as presented above, calculated using power analysis (G*Power) criteria; p <.05; 1-beta = .80; ES (between): d=0.15 - 0.30; 3 groups, 3 MZP. Required N=165 subjects, 55 per group.

Sample extraction: ad hoc/general sample; no other drawing is possible in this context.

Inclusion of protected groups not provided.

Inclusion criteria:

-age: 18 to 65

-Diagnosis of one of the following anxiety disorders: F40.1: Social anxiety disorder/social phobia; F41.0 Panic disorder with or without agoraphobia, F4l.1 Generalized anxiety disorder.

-sufficient knowledge of German

-Average intellectual capacity (assessment during te- lephonic/video screening).

-Sufficient time for the weekly sessions and homework assignments.

-Acceptance of the study protocol

Exclusion Criteria:

-Participation in psychotherapeutic and/or psychological treatment or counseling, as well as participation in other psychological group services, planned at the same time or in the following three months.

-Symptoms of major depression, bipolar affective disorder or mania (current as well as history), psychotic, schizophrenic and/or schizo-affective disorders, chronic grief reaction, severe anorexia or bulimia, substance dependencies (alcohol, illicit drugs).

- severe personality disorders, especially borderline, narcissistic, anti/dissocial and paranoid personality disorder
- acute suicidality
- Switch, dose change, or complete discontinuation of psychophar- maka in the past or following three months.

  Lack of decision making ability

3.6. Is consent obtained from participants (or their legal representative, if applicable)?

Yes (attach consent form)

No

If no, why not:

3.7. Proximity or dependency relationship between participants and investigators (e.g. student-lecturer, employee-employer, etc.). Is voluntariness guaranteed in this context?

There is no dependency relationship

3.8. How vulnerable are the participants from the submitter's perspective?

The test persons are people with increased psychological distress (social anxiety disorder, generalized anxiety disorder, panic disorder); accordingly, they are more vulnerable than psychologically normal people, but less vulnerable than people with severe psychotic, affective and/or personality disorders, who are excluded from the study. In this respect, their clinical vulnerability level is to be classified as medium.

3b. Data protection

3.9. What personal data is collected?

Name, place of residence, e-mail address, telephone number, age, gender, marital status, education, nationality, psychometric data from clinical procedures, past psychotherapy(s), psychiatric treatment, intake of Psychotropic drugs

3.10. How should the anonymity of the participants be guaranteed?

Name, e-mail address and telephone number are required for organizational purposes and for sending the links for the online data collection. These data are recorded independently of the other - study-related - data only by the study director in an Excel spreadsheet and stored in a password-protected laptop. No other persons have access to it. All other data will be recorded pseudonymously via a trial perso- nence code to be generated by the patients themselves (= individual code).

3.11. If complete anonymization is not possible, how is privacy protected?

Anonymity is to be protected on different levels. 1. At the level of the practitioner, it is protected by the professional laws. The therapists are subject to the confidentiality obligation according to the Austrian Psychologists' Act and the German Psychotherapists' Act. Accordingly, no information about the persons and their treatment may be disclosed to the outside. Only in the course of supervision and intervision may the contents of the units be discussed anonymously. However, the supervisors are also subject to the same professional laws as the therapists, so that the subjects' data is also protected at this level.

At the level of the research data, it is not possible to assign the data to a specific person due to the pseudonymization using individual codes; unless the person discloses their individual code.

On the level of the concrete personal data, anonymity is ensured by the fact that only the study director has access to these data and only he/she is aware of them; however, he/she is not able to assign the names to the data, as he/she does not have the individual codes. The personal data are accessible only to the study director through a password-protected table in an equally protected laptop.

3.12. How is it possible for participants to view their personal data?

Participants can only request access to their personal data from the study management by disclosing the individually issued code. Individual information and insight into the data and the individual results of the study can only be provided after the study has been completed in individual feedback sessions. For this purpose, however, the subjects must submit a request to the study director.

3.13. Can participants find out about the research findings?

■ Ja No If no, reason:

3.14. How and for how long can participants request the deletion of their data?

Upon request and notification of the individual participant's code, the data can be deleted. The deletion of the data can be requested until the end of the data collection. Afterwards, they are stored in a password-protected computer for further analyses and re-analyses.

3.15. The data processing takes place:

personal, justification:

Indirectly personal

How is anonymization performed?

Pseudonymization takes place via individually generated codes according to a six-digit key.

3.16. Are voices, sound, images or videos recorded?

☐ Ja No

If yes: Consent of participants to be recorded? Yes

3.17. What are you doing to ensure that the participants from the study are be able to opt out or withdraw their own data?

Already in the run-up and in connection with the recruitment, the participants are informed that they can withdraw or terminate their participation in the study at any time by means of a letter (e-mail) to the study management. This will exclude them from the study with immediate effect. At the beginning of the study, the participants will be given a

consent form is submitted or sent by e-mail (see appendix), in which this procedure and all other information about the study are described. If the withdrawal is associated with a request for deletion of the individual data, the individual code must be disclosed to the study management when communicating (e-mail).

3.18. How will the data be stored and/or destroyed after the project is completed?

The data are stored electronically and kept in an encrypted ord- ner of a password-protected laptop in pseudonymous form. The data are stored for 30 years. The data will be fully anonymized and used for further research. In the consent form, the participants are informed about the data processing and agree to it with their signature.

3c. Consequences for participants

3.19. Risk and consequence assessment (e.g., pain, inconvenience, injury to personal integrity, and measures to avoid and/or provide for unforeseen/undesirable events).

In principle, undesirable side effects can occur during psychotherapy (Linden, & Strauß, 2018). The occurrence of undesirable side effects cannot be ruled out in the treatments planned here. However, the primary goal of the treatments is to reduce anxiety and general psychopathological symptoms and to improve general well-being and quality of life. According to the knowledge of previous studies, the risk of unpleasantness and negative developments in the field of PPT and KVT can be considered low. Cognitive behavioral therapy is the treatment of choice for anxiety disorders and has shown very good effects to date. Positive Psychotherapy aims to strengthen the resources and strengths of the participants. In the run-up to the treatment, the participants are informed that they should inform their therapists at any time if undesirable effects should occur. If the negative changes should not be reduced, the study management should be contacted by the patients or treatment providers.
In addition, participants are given the telephone number of the crisis service responsible for their area. If there are repeated complaints about adverse experiences or side effects of the therapies, a corresponding procedure for the systematic recording of such events will be implemented at the end of the treatment (T2) (e.g. INEP, Ladwig et al., 2014).

3.20. What measures are taken to prevent risks?

The therapists are psychologists (Master's degree) who have extensive theoretical and practical experience in the field of clinical psychology and psychotherapy. In addition, the therapists are familiarized with the respective manuals and trained in them. All therapy sessions are recorded to ensure fidelity. All therapists receive regular super- vision and conduct intervision. In the run-up to the study, the nearest crisis services are recorded and the participants are given their telephone numbers and contact details. In addition, they are informed that they will have professional contact persons throughout the entire period of the study in the treatment providers and the study and project management. All participants will be given their contact details. Before the start of the intervention, it is also determined whether the participants are suicidal or suffering from severe depression. Both are exclusion criteria for participation in the study. The therapists are given extensive information on how to proceed in an emergency situation (crisis, suicidality).
You will also receive appropriate checklists in their treatment manuals
The participants are provided with a set of guidelines to help them react appropriately in the event of a crisis. In the event of acute suicidal tendencies, an individual emergency plan is first drawn up with the participants and the duty of confidentiality is enforced.
The participants will be informed about the cancellation of § 37 of the Psychology Act (this will be signed by the participants in the course of the declaration of consent), and the therapists will turn to the study and/or project management, with whom possible solutions will be worked out.

3.21. Anticipated benefits or potential benefits WiFor the included participants.

Based on existing research, treatment (both PPT and CBT) is expected to result in a significant reduction in anxiety and general psychological symptoms and negative states of mind (Laireiter & Furchlehner, 2018; Fava et al., 2005). At the same time, a strong increase in positive sensitivities, well-being, and life satisfaction is expected, especially in the positive-psychological treatment conditions. Another benefit of participating in the study is that the participants will receive the treatments free of charge, which is also a great benefit in view of the sometimes very long waiting times for professional help.

3.22. Methods to locate, record, and report adverse effects (Describe when, by whom, and how,
z. e.g. free questioning and/or on the basis of lists)

During the weekly therapy sessions, undesired effects or side effects of the treatments can be immediately recognized by the therapists. The therapists are required to document not only the course of the session and the interventions, but also the perceived effects and feedback from the participants (especially negative and problem-related). In the case of communication of a negative development, this must be documented in particular; if the negative development is classified as problematic or dangerous, an individual discussion must be held promptly with the participant concerned. If no adequate solution can be found, the study and/or project management should be contacted in order to work out a solution (see also above). In addition, the intervision and the supervision of the therapists offer space to reflect on possible problems and problematic developments in individual participants and to obtain professional advice from trained clinical psychologists/psychotherapists. In addition, in the event of stress or undesirable developments outside or between treatment sessions, patients are able to contact the treatment providers at any time by e-mail or contact the study or project management.

3.23. If necessary: Plan for treatment and/or care after individuals have completed their participation in the project (who will be responsible and where).

If problems or negative effects occur after the end of the treatments - in the follow-up period or afterwards - the participants are asked to contact the study or project management (but also the treatment providers can be contacted). If necessary, the participants can take advantage of a counseling session with these persons. The aim is to clarify the problems and work out individual solutions. It should also be determined whether the problems can be traced back to the treatment previously completed. In any case, the conversation should be documented. The solutions to the problems mentioned could include support for the person concerned in seeking help from a psychotherapist in the region, visiting their family doctor or a specialist themselves, or - in the worst case - making use of a crisis intervention center or medical facility, provided there is an indication for this. However, if necessary, the study or project management could also offer a further supportive intervention.

will be. Both are trained psychotherapists or psychotherapists in training.

3.24. Amount and procedure for the remuneration of participants (amount as well as wollir this is paid; e.g. travel expenses, loss of income, etc.).

No remuneration of the participants is foreseen; the participants receive free psychological treatment as indirect and immaterial remuneration (see above).

3.25. Amount and procedure for compensating participants (amount of payment and what it is paid for; e.g., pain, etc.)

The participants do not receive any compensation; no aversive stimuli are applied or severity caused.

3d. Other ethical aspects

3.26. Are the participants fully informed about the nature, objective and content of the project informed?

Yes No, reason:

3.27. Are participants being deceived?

Yes No

If yes, description and justification:

3.28. Ethical Considerations: Identify and describe any issues that may arise.

Since the practitioners are (only) in training to become clinical psychologists or psychotherapists and have not yet completed this training, they may lack a full range of experience and a broad repertoire of methods of psychological treatment to deal with all the problems that may arise. This can be compensated by the following components:

1. fully formulated and detailed manuals,
2. an intensive enrollment with self-experience,
3. an accompanying continuous intervision, if necessary in the presence of the study leader or - if desired - the project leader
4. an accompanying regular supervision by clinically experienced experts in PPT and/or CBT,
5. the ability to have practical experience from their own internships and professional activities in psychiatry or psychosomatics and the treatment of outpatients within the framework of their training, and
6. having sufficient knowledge in the field of clinical psychology, psychotherapy and positive psychology.

It could happen that practitioners feel overwhelmed in the course of the intervention; this should be recognized quickly in the context of intervision and/or supervision and an adequate individual solution found (e.g. change of one or both practitioners). In the event of a change of therapist, the patient's continued care is ensured in good time. In the event of a change, another practitioner, who has also been intensively trained in the manuals, takes over the treatment. In this case, a controlled handover takes place.

In addition, all practitioners are selected according to the following strict criteria. These are:

- completed master studies in psychology
- Currently in training to become a psycho1ogical psychotherapist or clinical psychologist
- Clinical experience in the field of psychiatry/psychosomatics with adults
- Positive psychology lectures or seminars taken in the course of study
- Sufficient time to be able to hold regular sessions (min 6hrs per week)

If, despite comprehensive screening and initial diagnosis, a suicidal crisis does occur, the patient may be referred and/or crisis intervention initiated.

The impression could also arise that psychotherapy is being offered with this study. In order to avoid this impression, the participants are informed from the beginning that this project is not a (statutory) psychotherapy or a psychological treatment, which is carried out by a psychotherapist in private practice and which is not covered by the health insurance.

The participants sign the declaration of consent and acknowledgement of this in the consent form. The participants sign the information and acknowledgement of this in the declaration of consent.

3.29. What is the relationship between potential risks of the study and expected benefits?

In our opinion, the expected benefits of participation in the study clearly outweigh the risks. Both the participants (reduction of anxiety symptoms and improvement of general well-being), the research (important findings in a young field of research), and the

society (new mechanisms of action to increase well-being are being evaluated) benefit from this project.

3.30. According to the risk plan, when is the project to be suspended? Under which Under what circumstances is the project cancelled?

If at least 10% of the participants show negative effects, the treatment is interrupted. If more than 15% report a deterioration, the study will be interrupted immediately and the cause will be investigated. If the study is discontinued, the participants will be supervised by experienced psychotherapists in an individual session and, if necessary, they will be supported in finding further treatment/psychotherapy.

3.31. Is insurance required?

Yes No

If yes: Insurance company

4. other comments

The created manuals correspond to the standard of psychological treatment manuals and were derived from existing and evaluated manuals or represent elaborations or abbreviations of existing manuals. In addition, they were reviewed by experts in PPT and KVT and found to be suitable.

5. project team

Indicate all staff members involved in the project:

Name	Institution	Function*	Qualification**
a.o. Univ. Prof. Dr. Anton Rupert Lai-reiter	Department Psy-chology, University of Salzburg	Project Manager***	a.o. Univ.-Prof. Clinical psy-chologist, psy-chotherpeut; Advanced training in Positive psy-chology
Catiana Luisa En-gelhardt	Department of Psy- chology, University of Salzburg	PhD student, preparation of ma-nuals, planning and evaluation of the study.	Psychologist (MSc), doctoral studies at the University of Salzburg, Psychological Psychotherapist

Mag. Brigitte Schweiger-Schrader	Department of Psychology, University of Salzburg	Supervisor	Clinical psychologist, teaching commissioner and trainer in positive psychology
N.N.		Supervisor	Trainer in Positive Psychology

*z. e.g. planning, evaluation, management, data collection
** e.g. Prof., Senior Scientist, Dissertantln, Postdoc
*** Project leader should have a PhD in the research field

Are there conflicts of interest of research staff involved?

☐ Ja No

If yes: Fill out and attach conflict of interest supplementary sheet

6. name and signature of the applicant

Name: Catiana Luisa Engelhardt
Institution/Company: Department of Psychology, University of Salzburg

Position: PhD student

Applicant's signature: I hereby certify that the information provided in this application is correct and that I believe that the implementation of the project will be possible in accordance with national regulations and with the principles of good scientific practice.

Signature of the applicant, date

If the project is a dissertation (PhD thesis) or master's thesis or diploma thesis **Name and signature of the supervisor**

Name: retired Univ.-Prof. Dr. Anton-Rupert Laireiter
Institution/Company: Department of Psychology, University of Salzburg
Position: retired university professor, private lecturer. Main focus among others: Psychotherapy, Positive Psychology

Supervisor's signature: I hereby certify that the information provided in this application is correct and that I believe that the project will be carried out in accordance with national regulations and with the principles of good scientific practice.

Signature of the supervisor, Date

To be enclosed:

- Project application
- Study participant information/consent form
- Brief academic resume/qualifications of the applicant(s).
- Conflict of interest supplement (if applicable)
- Existing ethics committee votes (if applicable).
- Participant insurance (if necessary)
- Questionnaires, interviews, etc. used in the study for data collection.
- Advertisements, brochures, etc. for recruiting participants
- Check list
